# *Laminaria digitata* and *Palmaria palmata* Seaweeds as Natural Source of Catalysts for the Cycloaddition of CO_2_ to Epoxides

**DOI:** 10.3390/molecules24020269

**Published:** 2019-01-12

**Authors:** James W. Comerford, Thomas Gray, Yann Lie, Duncan J. Macquarrie, Michael North, Alessandro Pellis

**Affiliations:** Green Chemistry Centre of Excellence, Department of Chemistry, University of York, York YO10 5DD, UK; Thomas.Gray.1996@hotmail.com (T.G.); yl2864@york.ac.uk (Y.L.); duncan.macquarrie@york.ac.uk (D.J.M.); michael.north@york.ac.uk (M.N.); ale.pellis@york.ac.uk (A.P.)

**Keywords:** CO_2_ sequestration, green catalyst, clean synthesis, sustainability

## Abstract

Seaweed powder has been found to act as an effective catalyst for the fixation of CO_2_ into epoxides to generate cyclic carbonates under solvent free conditions. Model background reactions were performed using metal halides and amino acids typically found in common seaweeds which showed potassium iodide (KI) to be the most active. The efficacy of the seaweed catalysts kelp (*Laminaria digitata*) and dulse (*Palmaria palmata*) was probed based on particle size, showing that kelp possessed greater catalytic ability, achieving a maximum conversion and selectivity of 63.7% to styrene carbonate using a kelp loading of 80% by weight with respect to epoxide, 40 bar of CO_2_, 120 °C for 3 h. Maximizing selectivity was difficult due to the generation of diol side product from residual H_2_O found in kelp, along with a chlorinated by-product thought to form due to a high quantity of chloride salts in the seaweeds. Data showed there was loss of organic matter upon use of the kelp catalyst, likely due to the breakdown of organic compounds and their subsequent removal during product extraction. This was highlighted as the likely cause of loss of catalytic activity upon reuse of the Kelp catalyst.

## 1. Introduction

An increase in net global CO_2_ emissions is expected to have significant consequences over the next few decades [1,2,3,4,5]. The majority of these emissions are a result of fossil fuel combustion for transportation and power generation, for domestic homes, industry, chemical processes (such as the Haber-Bosch) and production of meat [6,7,8,9]. A reduction in global CO_2_ emissions is paramount, however the complexity of this issue requires a holistic approach to tackle this significant problem. Whilst the production of chemicals from CO_2_ is not the answer alone, use of flue gas waste streams will allow the utilization of CO_2_ as a benign C1 feedstock to produce more sustainable chemical compounds. Production of five membered cyclic carbonates from the cycloaddition of CO_2_ to epoxides is one of the leading examples of this practice. The synthesis of ethylene and propylene carbonate [10] has received much attention in recent years due to their use as electrolytes in lithium ion batteries, the production of which has increased rapidly in parallel with increased production of electric cars. Furthermore, cyclic carbonates can be used in the synthesis of polymers [11,12,13], as intermediates in the production of dimethyl carbonate [14,15] and even as green polar aprotic solvents aimed at replacing traditional solvents such as DMF, DMSO, NMP and acetonitrile [16,17]. There have been many different catalysts reported for this synthesis each with their individual benefits and drawbacks. Aluminum complexes reported by North [18] and Kleij [19] are extremely active in that they are able to catalyse the cycloaddition of CO_2_ under ambient temperatures and pressures. Although these reaction conditions are impressive, the multistep synthesis of the catalysts tend to have poor E-factors, leading to increased use of resources and energy, consequently increasing the carbon footprint for the process and overall environmental impact. Alternatively, metal halide salt catalysts tend to be much cheaper, but often use high temperatures and pressures of > 100 °C and 20–60 bar, often along with the necessitated addition of a hydroxyl containing co-catalyst to aid stabilization of the alkoxide intermediate and drive the reaction to high conversions. Interestingly, there have been a number of natural organic compounds reported as effective co-catalysts, such as cellulose [20], β-cyclodextrin [21], lecithin [22] and betaines [23]. Various amino acids have been reported as very effective co-catalysts, with histidine being one of the most active [24]. One of the most effective metal halide salts reported to date is potassium iodide (KI). Typically, this is produced from potassium hydroxide and iodide, where potassium is either mined from underground ore deposits, salt lakes or brines, and the iodide can be sourced from either caliche ore, brines or seaweeds (along with potassium salts), each often having direct environmental consequences [25,26,27]. Further to this, extraction, isolation, purification and transportation of such species require huge amounts of energy (the average mine consumes 25 MW/h per tonne of material processed [28]), having an indirect environmental impact.

This paper details an investigation of the use of two different seaweeds as one-component, natural catalysts for the formation of cyclic carbonates due to the high quantities of metal halides found in the plant along with amino acids in the form of proteins. Direct use of the plant with minimal preparation has a significant number of benefits; the plant naturally sequesters inorganic minerals out of the sea removing the need for refining and purifying metal salts or halogens, seaweed is sustainable and extremely abundant, it is accessible and cheap throughout the world, along with the benefit of having reduced operator and environmental risk with regards to handling and manipulation. Kelp was chosen due to the well reported high concentrations of iodide and salts incorporated within the plant [29,30,31] and dulse was chosen as a comparison. Both contain amino acids, proteins and polysaccharides (such as cellulose, alginate, fucoidan and carrageenan) that could be beneficial to the synthesis [32]. Aside from being edible, both seaweeds already have industrial non-food applications with established harvesting and processing routes. They are commonly used as organic fertilizers and soil conditioners (both today and historically) [33], for the production of seaweed gums for use in the pharmaceutical, medical and cosmetics industries (extraction of alginates from *Laminaria digitata*, along with agar and carrageenan from *Palmaria palmata*) [34] as well as use in animal feed due to their protein content (up to 50% crude protein for red seaweeds and approximately 14% for the more easily harvested brown seaweeds) [35]. 

## 2. Results and Discussion

### 2.1. Conversion to Styrene Carbonate Using Model Catalyst and Co-Catalyst

Initially, a screen of different metal halides catalysts with different amino acid co-catalysts was performed as a baseline for comparison with the seaweed catalysts and also to identify any synergistic effect between the metal and amino acid co-catalyst. Full details of the procedures used are detailed in Experimental Section 4.3. Basic amino acids histidine and lysine were chosen for their reported ability to activate CO_2_ by formation of a carbamate salt [36], whereas glycine was chosen as a non-basic polar comparison, Table 1. Overall, the less electronegative potassium (K) salts were found to be more active than the sodium (Na) and calcium (Ca) equivalents. A small quantity of 1-phenyl-1,2-ethanediol by-product was observed, which was found to increase when incorporating polar amino acids histidine and lysine as co-catalysts, most likely due to residual water contamination, (SI, Table S1a–e). However, the addition of amino acid co-catalysts containing a basic group (histidine and lysine) significantly increased conversions to styrene carbonate for all metal halide catalysts containing a bromide and chloride counter ion; histidine was found to give the greatest synergistic effect of all, in accordance with the literature [37]. CaCl_2_ achieved 80.0% conversion to **2a** when used with histidine as opposed to being inactive when used alone. However, a very different effect is observed for systems using an iodide counter ion, where for KI, introduction of an amino acid actually decreased conversions to carbonate. This may be due to increased interaction between either the metal cation and/or iodide anion with the amino acids and therefore reducing desired interaction with reagents, giving reduced conversions. An impressive synergistic effect is seen with NaI and glycine achieving a conversion of 97.1% as opposed to 23.1% using NaI or no conversion using glycine alone. The reason for this observation is unclear, however this may be attributed to the increased ability of glycine to aid the disassociation of the NaI ion pair. Comparatively, the chloride and bromide sodium salts appear to be less affected by the polarity of the amino acids possibly due to increased lattice energy and metal electronegativity. In these systems, the ability of the histidine to act as a catalyst alone (41.5%) becomes much more important and has the dominant effect on total conversion to styrene carbonate.

The combination of various metal halides with amino acids has the potential to form very active catalytic systems. With the investigated metal halides likely to be present to some extent in seaweed, along with various amino acids side chains also being present in the form of proteins, two common seaweeds were investigated as catalysts for the synthesis of styrene carbonate.

### 2.2. Kelp and Dulse as Catalysts

A simple activity screen was performed in the first instance to identify if seaweed had catalytic potential. Kelp (*Laminaria digitata*, brown seaweed) and dulse (*Palmaria palmata*, red seaweed) were chosen for investigation due to the high concentrations of iodide previously reported for kelp and the excellent conversions obtained using iodide salts seen in the initial model screening. Both seaweeds were sourced from the North coast of Northern Ireland [38]. The kelp and dulse were blended and sieved prior to drying as detailed in Experimental Section 4.2, allowing us to maximise reproducibility of our investigations as well as understand the effect of particle size. Due to the many different organic and inorganic species likely to be contained within the seaweeds, a fixed 80% weight of seaweed catalyst relative to epoxide was used in the syntheses as detailed in Table 2. Reduced loading of the catalyst was found to also reduce conversions, (SI, Figure S1), whereas increased loading to 100% wt. caused issues with the reaction’s optimal mass transfer. Conversions to carbonate did not appreciably increase when using pressures over 20 bar, however, 40 bar was used for all investigations to be comparable with the initial metal halide study, (SI, Table S2). Overall, kelp was found to be more active towards the formation of styrene carbonate than dulse, achieving a 29.9–33.2% conversion to styrene carbonate in 3 h at 120 °C and 40 bar CO_2_; however, some unexpected effects were observed. In contrast to the standard metal halide comparison shown in Table 1, there was a notable quantity of by-product formed by both of the seaweed catalysts. 

**Scheme 1:**
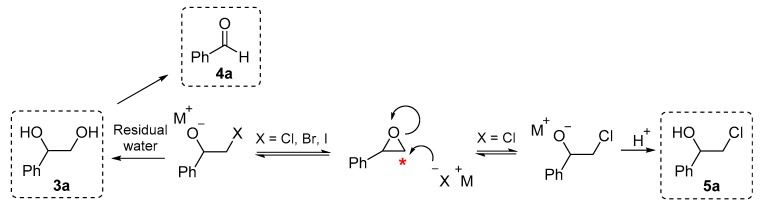
Formation of benzaldehyde, chloro and diol by-products.

**Scheme 2:**
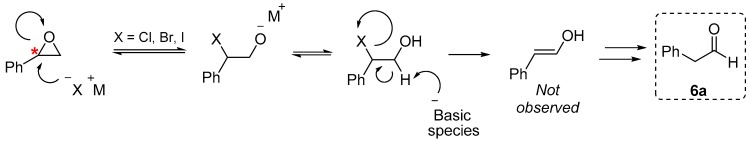
Formation of phenylacetaldehyde by-product.

Although a certain quantity of diol by-product **3a** was expected due to residual water, formation of 1-phenyl-2-chloroethanol (**5a**) was also found along with trace quantities of benzaldehyde (**4a**) and phenylacetaldehyde (**6a**) Scheme 1; Scheme 2. Formation of the chlorinated species **5a** is most likely due to the relatively high concentrations of chloride salts found within the seaweeds. Although bromide and iodide anions are nucleophilic enough to ring open the epoxide, they are large soft nucleophiles and as such, able to act as good leaving groups upon attack from an activated CO_2_ carboxylate electron pair. The increased electronegativity of the chloride makes this a less good leaving group but a relatively good nucleophile and as such, forms a relatively stable chlorinated by-product. Formation of benzaldehyde **4a** from diol by-product **3a** has been well reported [39,40,41] however, due to the large quantity of potentially active catalytic species within this system; the exact route of formation was not identified. Interestingly, in order to preserve the benzylic carbon, formation of **6a** suggests halide attack at the tertiary carbon, rather than at the less hindered secondary carbon.

Particle size was found to have a dramatic effect. Particles sizes <125 µm and between 300–500 µm achieved the best conversions, with the smallest particles being superior overall, and particle sizes between 125–300 µm gave significantly worse conversions, on average ~74–91% lower. The kelp synthesis was repeated in duplicate with the same trend being constantly observed achieving, 28.7% carbonate for <125 nm particle size, 3.3% for 125–300 nm and 33.0% for 300–500 nm. To explore this further, larger particle sizes >500 µm (which gave a good performance of 31.8% carbonate) were ground to 125–300 µm and then used in the synthesis, yet these were found to still give lower conversions of only 5.3% styrene carbonate. Although the reason for this is not immediately apparent, this observation may be attributed to stirring kinetics: particles of certain size are able to agglomerate, therefore significantly reducing reaction rates. This effect considered, each particle size was investigated herein for each of the subsequent studies.

### 2.3. Reproducibility Using Different Kelp Seaweeds

Due to the variation in catalytic activity found between kelp and dulse, a more detailed study was performed focusing on whether a single species of seaweed could give reproducible catalytic activity if sourced from a different location. Three samples of kelp were sourced from the Plymouth Marine Laboratory and were harvested between September and November 2017 from Broadsands Beach, Paignton, UK. Kelp was chosen for investigation due to its superior activity.

#### 2.3.1. Catalytic Activity

Each of the kelp samples (B to D) were dried and blended as previously described in Experimental Section 4.2. Identical reaction conditions and catalyst loading were used for the syntheses, with each of the different particle sizes investigated. Similar to previous observations, particle size had a significant effect, with seaweed particles sizes between 125–300 µm giving substantially lower conversions to styrene carbonate (for example, 75% lower conversion between < 125 nm to 125–300 nm particle sizes for kelp D), Table 3. Despite extensive drying of the seaweed prior to use, diol by-product of between 22–38% was still observed.

A number of drying techniques were employed, however TGIR analysis showed ~8% water by mass was retained in the seaweeds after treatment (SI, Figures S3 and S4). Kelp D was found to give the highest conversions, achieving 38.8 and 37.3% **2a** using particles sizes <125 µm and >500 µm respectively. Conversion to diol by-product **3a** from residual water was also high (79.2% for <125um kelp D) when compared to kelp B (60.4%) and C (60.9%; SI, Table S3). Interestingly, quantities of 2-chloro-1-phenylethanol (**5a**) remained relatively consistent across the series, on average 12.4% and similarly, minor by-products **4a** and **6a** also remained consistent giving an average of 0.5 and 1.9% respectively. Conversions to **2a** between kelp B and C were comparable except for the particle size > 500 µm for Kelp B, achieving very low conversions of only 6.9% **2a**. The ratio of carbonate **2a** to diol by-product **3a** was relatively consistent for all particle sizes except reactions using kelp 125–300 µm. This suggests formation of diol is independent to the formation of carbonate, where ring opening of the epoxide by water does not require any of (or access to) the catalytic species present in the seaweed. Kelp C showed very similar activity between the particles sizes of <125 µm and >500 µm of 26.4 and 25.6% conversion to **2a**. Despite the relatively good conversions, there was still variation in conversion to **2a** amongst comparable samples in the order of <125 µm kelp D (38.8%) superior to both < 125 µm kelp B (27.6%) and <125 µm kelp C (26.4%). In order to further understand the distinct variation in activity, ICP-MS analysis of metal content along with an amino acid profile was constructed for Kelp samples B–D.

#### 2.3.2. Metal Concentrations

ICP-MS analysis was performed externally and samples prepared as detailed in Experimental Section 4.10. Low concentrations of Li, Al, Fe, Cu, Zn, Sr and Ba were found in all of the kelp samples, Table 4. Each of the seaweed samples contained a high proportion of sodium, with kelp D containing the most at 4.86% wt., kelp C 4.39% wt. and kelp B 4.13% wt.; the opposite is true for calcium (D—1.33, C—1.45 and B—1.72% wt.). 

Kelp D showed the highest K content of 2.54% wt., with both kelp B and C having lower concentration of 1.54% and 1.45% wt. respectively. This correlates well with previous observations, in particular, the high activity of K demonstrated in the model screen (Table 1) and the increased activity of kelp D shown in Table 3. The distribution of mineral content across the series does not appear to be affected by harvest date, where kelps C and D were both sourced in November, yet have very different metal contents, whilst kelp B was harvested in September and has similar metal concentrations to kelp C. Whilst K content correlated well with activity, the amino acid profile and protein content of the kelp sample was also investigated.

#### 2.3.3. Amino Acid Profile and Protein Content

**Figure 1:**
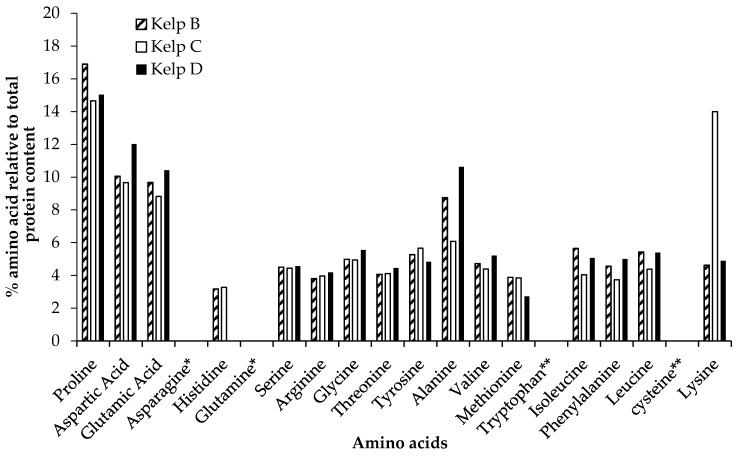
% weight amino acids relative to total protein content for kelp B, C and D. Asterisks against asparagine* and glutamine* denote conversion to aspartic and glutamic acid. Tryptophan** and cysteine** highlight an expected absence of these amino acids, as a result of the work up procedure and analytical method.

CHN analysis of kelp samples B–D showed the total protein content [42] to be 7.8% for kelp B, 7.8% for kelp C and 8.8% for kelp D by mass. The amino acid profile was performed using an established technique [43] detailed in Experimental Section 4.6. Asparagine, glutamine, tryptophan and cysteine are absent from the data as a result of the hydrolysis preparation for HPLC analysis, Scheme 2; though it should be noted that, aspartic acid and glutamic acid are proportional to asparagine and glutamine originally present in the kelp samples prior to hydrolysis and the amine precursor will be referred to throughout the discussion. Interestingly, the proportion of basic (containing a second amine group) amino acids is relatively high for each kelp sample (Figure 1). Proline, aspartic acid, glutamic acid, arginine and lysine made up between 45–51% of the total amino acid content (TAAC) of the seaweed proteins present in the kelp. Interestingly, only low quantities of histidine were found in kelp samples B and C, whilst being absent in kelp D. Proline, asparagine and glutamine were found to be the dominant amino acids for all kelp samples with kelp D containing the most of these amino acids, correlating well with catalyst activity. 

**Scheme 3:**
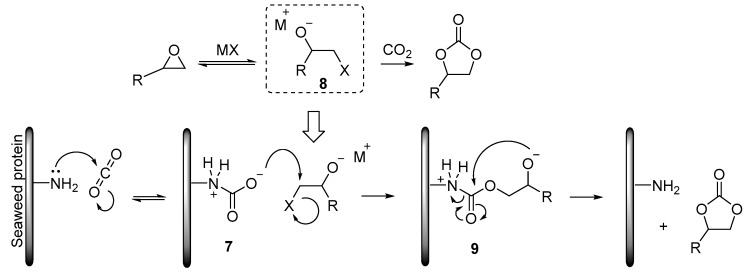
Catalytic cycle of a basic amino acid with metal halide.

Although it is unlikely that neutral amino acids proline and alanine would contribute significantly towards the catalytic activity found within the system, any of the basic amino acids (including carboxamides asparagine, glutamine as well as lysine) are likely to contribute towards the activation of CO_2_ (Figure 1). However, activity has been shown to be dependent on amino acid type (Table 1), where despite the relatively high ratio of lysine present in kelp C, this particular amino acid does not have high activity when used alone suggesting that the lower conversions seen with kelp C are more related to the concentration of other amino acids as well as metal halides. Nevertheless, mechanistic pathways have been suggested in the literature [36], where the basic amines are able to form a carbamate salt **7** with CO_2_, which is then able to subsequently attack halide activated alkoxide intermediate **8** regenerating the metal halide catalyst, Scheme 3. Cyclisation of intermediate **9** forms the cyclic carbonate product and the free amine co-catalyst.

### 2.4. Reuse of Kelp Seaweeds

Due to the proposed partially heterogeneous catalytic nature of this system, reuse of the kelp catalysts was investigated, Table 5. After first use for the synthesis of **2a**, each of the kelp catalysts was filtered from the reaction mixture, washed three times with deuterated chloroform for ^1^H-NMR analysis and dried in a vacuum oven overnight as detailed in Experimental Section 4.7. Prior to reuse in a second synthesis, the kelp catalysts were analyzed using TGA which showed that after one use, an average mass loss of 9.5% organics relative to residual inorganic material occurred. Reaction conditions used for the second use were identical to the first use syntheses, with the quantity of epoxide being adjusted to maintain an 80% wt. catalyst to epoxide ratio and account for loss of material during the isolation, analysis and washing stage. In all cases the second use conversions were significantly lower than conversions obtained during the first use, where kelp B and C conversions reduced by 76.8% and 71.6% with kelp D reducing by 58.0%. The higher reuse conversions obtained with latter may be attributed to kelp D having increased concentrations of K and TAAC relative to kelp B and C prior to use. Similarly, conversion to by-product **5a** also reduced by around 44–60% across the series, suggesting a loss of chloride ions from the system. An increase in ratio of **2a**:**3a** suggests further incorporation of water into the Kelp during the isolation and reuse process.

As none of the potential catalytically active species within the seaweed are covalently bound, it is likely that proteins, amino acids and metals are dissolved out of the seaweed polysaccharide structure by the epoxide, product or by-products throughout the reaction; CO_2_ gas expansion may aid this too. Furthermore, at reaction temperatures of 120 °C the seaweed proteins are likely to denature and depolymerize making the subsequent oligomeric species more soluble in the reaction mixture. Methods to circumvent the issue of leaching are not immediately obvious due to the nature of the raw seaweed material being used, however, subsequent isolation and reuse of the active species may be possible with further investigation.

### 2.5. Substrate Scope Using Kelp D as a Catalyst

The substrate scope of kelp D was investigated due to its superior activity over kelp B and C, Table 6. Reaction conditions used were identical to the previously performed experiments, as detailed in Experimental Section 4.5. Interestingly, 1,2-epoxydecane (**1e**) gave very poor conversions to **2e** with no conversion at all to by-products **4**–**5e**. This may be attributed to the low polarity of the substrate, where minimal quantities of the highly polar active species, such as metal halides, are able to dissolve into the reaction medium and instead are retained within the seaweed structure. Conversely, production of diol by-product appeared to be much more of a problem when using polar epoxides **1b**,**c**. Despite the relatively high quantities of **3c** by-product, conversions to **2c** were good achieving 42.2%. Similarly, conversions to **2b** were good (43.8%) despite the relatively high formation of **5b** of 27.4%. This may be attributed to the increased activity of the epoxide towards the OH or Cl, increasing delta positive character on secondary carbon of the epoxide and allowing increased rates of nucleophilic attack from either a halide or water. 4-Chlorostyrene oxide (**1d**) gave much lower conversions compared with **1a** most likely due to the deactivating effect of the p-Cl.

## 3. Conclusions

The initial model study supported previous observations that amino acids are able to offer impressive synergistic effects when used with a range of metals halides. However, in contrast to this KI was found to be superior to all model catalytic systems investigated when used without an amino acid co-catalyst. This study has shown that raw and (relatively) unprocessed seaweed can be used for the synthesis of cyclic carbonates and CO_2_. Of the two species investigated, kelp was found to contain a higher proportion of catalytically active species. Although there was distinct variation in the activity of the seaweed catalysts, this difference could not be correlated with either the location or date of harvesting. Despite both kelp C and D being harvested in November at the same location, the latter was found to be ~47% more active, attributed to the increased quantities of K and basic amino acids found within the seaweed structure. All seaweed samples suffered from the formation of diol by-product due to residual water being retained in the seaweed despite extensive drying. Moreover, a chloro functionalized by-product **5a** was also detected by GC/MS and ^1^H-NMR, typically accounting for 12% of total conversion along with small quantities of benzaldehyde (**4a**) and phenylacetaldehyde (**6a**) Each of these by-products has useful applications, particularly in the production of pharmaceuticals, fragrances and plastics. Further work should investigate the possibility of calcination of the seaweeds to remove all traces of water, yet avoid significant decomposition of the amino acid species. It should be noted that although the relative quantities of amino acids was determined via hydrolysis and HPLC analysis, the form of the amino acids during the reaction was not known (extent of protein denaturing, depolymerization etc).

Interestingly, particle size was found to have a significant effect on conversions with particle sizes between 125–300 µm giving consistently low conversions throughout the study for all seaweed samples. This was attributed to the stirring kinetics, where particle sizes between 125–300 µm are more able to agglomerate and restrict diffusion of reactant species. Use of a solvent, such as propylene carbonate, to allow high concentrations of catalyst to be used, yet maintain efficient stirring would help identify if agglomeration is the core problem.

Further investigation into the use of other seaweed types (non-edible as well as edible) sourced from locations across the globe would highlight the full potential seaweed has to offer as an entirely natural, benign and cheap catalyst for the synthesis of cyclic carbonates.

## 4. Experimental Section

### 4.1. Chemicals and Equipment

KI, KBr, NaI, CDCl_3_, histidine, glycine, lysine, glycidol and 2-(4-chlorophenyl)oxirane were purchased from Sigma Aldrich Company Ltd. (Dorset, UK). NaCl, 1,2-epoxydecane, and epichlorohydrin were purchased from Fischer Scientific (Leicestershire, UK). NaBr and styrene oxide were purchased from ACROS Chemicals (part of Thermo Fisher Scientific, Leicestershire, UK). Kelp and dulse listed in Table 1 were purchased from Irishseaweeds.com and was sourced from the Northern Irish north coast. kelp samples B, C and D were kindly supplied from Plymouth Marine Laboratory (Plymouth, UK), harvested from Broadlands Beach and Elberry Cove in Paignton.

Nuclear magnetic resonance spectroscopy was carried out using a JEOL 400 MHz instrument (JEOL, Tokyo, Japan) in deuterated chloroform. TG-IR was carried out using a STA409 system (Netsch, Selb, Germany) linked to a gas cell in an Equinox 55 Infra-red spectrometer (Bruker, Billerica, MA, USA) by a heated gas line. Amino acid content was determined using an Infinity 1200 HPLC system (Agilent, Santa Clara, CA, USA). ICP-MS analysis was performed at the University of Edinburgh on an Elan 6100 DRC instrument (Perkin-Elmer, Waltham, MA, USA) and an Element 2 system (Thermo-Finnegan, Waltham, MA, USA). GC/MS data was obtained on a JEOL JMS-T200GC AccuTOF (JEOL) and Agilent 7890B GC (Agilent). ESI was performed on a Bruker compact time of flight mass spectrometer (Bruker).

### 4.2. Seaweed Preparation before Analysis and use as a Catalyst

The pre-dried raw seaweed was cut into ~2 cm^2^ squares and then blended. The blended seaweed was sieved in order to separate particles by their sizes: <125 µm, 125–300 µm, 300–500 µm, and >500 µm. These samples were then dried in a vacuum oven at ~60 °C for 24 h and then freeze dried before storage in a desiccator.

### 4.3. Standard Synthesis of Styrene Carbonate Using Metal Halides and/or Amino Acids

Styrene oxide (0.57 mL, 5 mmol) was added to a vessel within the multipoint reactor. Either a metal halide, an amino acid, or a combination of the two was added to the vessel with each component being added at 2 mol % with respect to styrene oxide. The vessel was then pressurized to 40 bar of CO_2_ for 3 h at 120 °C with stirring. The reactor was then submerged in liquid nitrogen until the temperature within the reactor had reached ~10 °C, at which point the pressure within the reactor was released. The reaction mixtures were then extracted and analyzed by ^1^H-NMR spectroscopy to calculate the conversions.

### 4.4. Standard Synthesis of Styrene Carbonate Using Seaweeds Kelp or Dulse

Styrene oxide (0.57 mL, 5 mmol) was added to a vessel within the multipoint reactor. 80% wt. relative to epoxide of either kelp or dulse was added to the reactor having been prepared as stated in Experimental Section 4.2. The vessel was then pressurized to 40 bar of CO_2_ for 3 h at 120 °C with stirring. The reactor was then submerged in liquid nitrogen until the temperature within the reactor had reached ~10 °C, at which point the pressure within the reactor was released. The reaction mixtures were then extracted and analyzed by ^1^H-NMR spectroscopy to calculate the conversions.

### 4.5. Synthesis of Different Carbonates Using Kelp D

Five mmol of four different epoxides was added to separate vessels within a multipoint pressure reactor. Kelp sample B, particle size <125 µm was added to the vessel at a loading of 50% weight with respect to the mass of epoxide. The vessel was then pressurized to 40 bar of CO_2_ for 3 h at 120 °C with stirring. The reactor was then submerged in liquid nitrogen until the temperature within the reactor had reached ~10 °C, at which point the pressure within the reactor was released. The reaction mixture was then extracted by dissolving in deuterated chloroform followed by filter paper separation of the seaweed and the reaction solution. The remaining oil was then analyzed ^1^H-NMR spectroscopy to calculate conversions.

### 4.6. Amino Acid Profile

Hydrolysis of the kelp samples was done as follow: 10 mg of algae were suspended in 25 mL of 6M HCl with 1% *w*/*v* phenol and heated at 150 °C for 30 min in a CEM Discover microwave (CEM, Buckingham, UK). The resulting mixture was filtered and evaporated under reduce pressure. The resulting solid was re-suspended in 30 mL of suspension solution consisting of Eluent A:MeOH:ISTDsol (5:4:1). The suspension was sonicated for 10 s and filtrated through 0.22 µm Whatman filters. An Agilent Infinity 1200 HPLC system equipped with a Poroshell 120 EC-C18 4.6 × 100 mm, 2.7 μm diameter column was used to perform the chromatographic separation. Temperature of the oven was set at 45 °C, a quaternary pump (G7111B) was used to pump eluents through the column with a pressure limit of 600 bar and a flow set at 0.4 mL/min. A DAD detector (G7115A) was set at wavelength 263 nm (FMOC) and 338 nm (OPA). Settings of the sampler (G7129A) were the following: draw speed-200 μL/min, eject speed-400 μL/min.

### 4.7. Reusability Study

Kelp sample B, C or D, of particle size <125 µm was added to a vessel in a multipoint pressure reactor. These Kelp samples had already been used as a catalyst once before and had been washed in chloroform and subsequently dried in air prior to reuse. Styrene oxide was added at such a quantity that Kelp loading was 80% weight of styrene oxide. The vessel was then pressurized to 40 bar of CO_2_ for 3 h at 120 °C with stirring. The reactor was then submerged in liquid nitrogen until the temperature within the reactor had reached ~10 °C, at which point the pressure within the reactor was released. The reaction mixture was then extracted by dissolving in deuterated chloroform followed by filter paper separation of the seaweed and the reaction solution, followed by ^1^H-NMR analysis.

### 4.8. Pressure Study

Method as with 4.3, except with different CO_2_ pressure of 10, 20, 30 and 40 bar.

### 4.9. Thermal Gravimetric Infrared Spectrometry (TGIR)

Approximately 50 mg of kelp sample was used and placed under nitrogen with a flow rate of 100 cm^3^ min^−1^. The sample was then placed under vacuum after being backfilled with nitrogen twice and heated to 500 K at a ramp rate of 2.5 K min^−1^.

### 4.10. ICP-MS

ICP-MS was run as a service by Dr Lorna Eades, ICP Facility Manager, University of Edinburgh (Edinburgh, Scotland). 400 mg of kelp sample B–D was taken and calcined at 450 °C for 2 h and then left in a desiccator to cool. 10 mg of the residual sample was digested in 2 mL ultrapure 70% nitric acid (≥99.999%) and diluted to a total volume of 100 mL using ultrapure water. 10 mL of each sample solution was sent for ICP-MS analysis.

### 4.11. ^1^H-NMR Conversion Calculation

Conversions were calculated using integrals obtained from ^1^H-NMR spectra. An example ^1^H-NMR spectrum of the conversion to styrene carbonate using kelp B 300–500 nm particle size, as shown in Table 3, can be seen in Supplementary Information Figure S5. A typical calculation is also shown, where the conversion of any particular species is found by dividing the integral with the sum of the integrals for all species.

## Figures and Tables

**Table 1:**
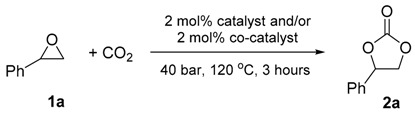
Conversion to styrene carbonate using different metal halides and amino acid co-catalysts.

Amino Acid Co-catalyst	Conversion to 2a Using Metal Halide Catalyst ^1^								Basic Sites ^2^
	KI	KBr	KCl	NaI	NaBr	NaCl	CaCl_2_	None	
**Histidine**	75.2	76.3	61.7	49.8	67.2	77.5	80.0	39.1	1 (2°)
**Lysine**	63.9	44.1	8.0	56.2	58.8	12.1	18.7	9.7	1 (1°)
**Glycine**	79.5	10.2	0.8	97.1	41.2	0.0	11.8	0.0	0
**None**	100.0	0.9	1.0	23.1	2.8	1.0	0.0	0.0	0

(1) 2 mol% of metal halide catalyst and/or 2 mol% of amino acid co-catalyst used in the synthesis. (2) 2° refers to secondary amine, 1° refers to primary amine.

**Table 2:** Conversion to styrene carbonate using kelp and dulse of different particles sizes.

Seaweed	Particle Size (µm)	Carbonate 2a (%)	Diol 3a (%)	BA ^1^ 4a (%)	CPE ^2^ 5a (%)	PAA ^3^ 6a (%)	Total Conversion from 1a (%)	Selectivity (%)
Kelp	<125	29.9	47.9	0.6	10.5	1.8	90.1	33.2
	125–300	2.9	59.0	0.3	12.9	0.8	75.6	3.8
	300–500	33.2	23.9	0.7	9.0	1.7	67.8	49.0
Dulse	<125	19.4	26.4	0.4	5.2	0.8	52.2	37.2
	125–300	4.3	25.1	0.4	4.9	0.6	34.8	12.3
	300–500	16.2	14.0	0.5	4.4	1.0	35.5	45.7

Aside from catalyst loading, reaction conditions used were identical to those used in Table 1, see Experimental Section 4.4. (1) BA—Benzaldehyde, (2) CPE—2-Chloro-1-phenylethanol, (3) PAA—Phenylacetaldehyde.

**Table 3:** Conversion to styrene carbonate using different kelp seaweed samples.

Kelp	Particle Size/µm	Carbonate 2a (%)	Diol 3a (%)	BA 4a (%)	CPE 5a (%)	PAA 6a (%)	Total Conversion from 1a (%)	Selectivity (%) ^3^
Kelp B (18/09/17) ^1^	<125	27.6	26.2	0.5	12.3	2.0	68.1	40.5
	125–300	8.4	24.0	0.5	11.3	1.7	45.4	18.5
	300–500	19.9	22.2	0.5	9.8	2.1	53.6	35.3
	>500	6.9	38.0	0.6	15.6	1.9	62.4	11.1
Kelp C (15/11/17) ^1^	<125	26.4	27.4	0.5	12.2	1.7	67.6	39.0
	125–300	5.9	30.5	0.5	13.0	1.5	50.9	11.6
	300–500	21.7	23.8	0.5	10.9	1.8	58.2	37.2
	>500	25.6	32.7	0.4	13.2	3.1	74.7	34.4
Kelp D (20/11/17) ^1^	<125	38.8 (63.7) ^2^	35.2 (28.2)	0.6 (0.1)	11.3 (8.1)	1.6 (0.07)	86.9 (100)	44.6 (63.7)
	125–300	9.4	37.5	0.6	15.4	1.5	63.7	14.7
	300–500	30.2	27.7	0.6	11.4	1.5	70.8	42.6
	>500	37.3	36.2	0.3	12.6	2.5	88.6	42.1

(1) Date that the seaweed was harvested. (2) Conversions when scale up to 20 mmols using a single Parr reactor. Full data showing conversion overtime given in SI, Figures S2 and S3) Selectivity towards carbonate 2a relative to total conversion from epoxide 1a.

**Table 4:** Metal concentrations in kelp seaweed samples B–D.

Kelp	Harvest Date	Metal Concentrations (mg g^−1^)											<125 µm Conversion to 2a (%)
		Li	Na	Mg	Al	K	Ca	Fe	Cu	Zn	Sr	Ba	
**B**	18/09/17	0.02	41.3	8.5	0.22	15.4	17.2	0.11	0.13	0.67	1.36	0.06	27.6
**C**	15/11/17	0.02	43.9	10.4	0.27	14.5	14.5	0.10	0.09	0.33	1.17	0.09	26.4
**D**	20/11/17	0.02	48.6	10.3	0.36	25.4	13.3	0.21	0.12	0.26	1.13	0.07	38.8

**Table 5:** Effect of reuse on kelp activity in the synthesis of styrene carbonate.

Kelp	Use	Carbonate 2a (%)	Reduction in Activity (%) ^1^	Diol 3a (%)	Ratio 2a:3a	BA 4a (%)	CPE 5a (%)	PAA 6a (%)	Selectivity (%)
**B**	1st	27.6	-	26.2	1:0.95	0.5	12.3	2.0	40.5
	2nd	6.4	76.8	21.9	1:3.47	0.4	5.5	0.9	18.4
**C**	1st	26.4	-	27.4	1:1.04	0.5	12.2	1.7	39.0
	2nd	7.5	71.6	23.7	1:3.15	0.4	6.6	1.0	19.4
**D**	1st	38.8	-	35.2	1:0.91	0.6	11.3	1.6	44.6
	2nd	16.3	58.0	20.3	1:1.25	0.8	6.5	1.2	44.2

(1) Expressed as a percentage relative to the conversions achieved during the first use of the kelp catalysts.

**Table 6:**
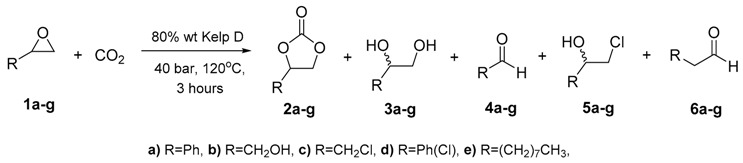
Substrate scope using kelp D as a catalyst.

Epoxide	2a–g (%)	3a–g (%)	Ratio 2a–g:3a–g	4a–g (%)	5a–g (%)	6a–g (%)	Total Conversion from 1a (%)	Selectivity (%)
1a	38.8	35.2	1:0.91	0.6	11.3	1.6	86.9	44.6
1b	43.8	28.7	1:0.66	0.03	27.4	0	100	43.8
1c	42.2	44.7	1:1.06	0.0	13.0	0	100	42.2
1d ^1^	20.6	20.8	1:1.01	0.4	0.6	0.4	54.6	37.7
1e	0.5	8.6	1:18.5	0.0	0.0	0.1	9.1	5.1

(1) 4-Chlorostyrene oxide.

## Supplementary Materials

The following are available online, Table S1a: Conversions to styrene carbonate and diol by-product using metal halides and histidine co-catalyst, Table S1b: Conversion to styrene carbonate and diol by-product using metal halides lysine co-catalyst, Table S1c: Conversion to styrene carbonate and diol by-product using metal halides glycine co-catalyst, Table S1d: Conversion to styrene carbonate and diol by-product using metal halides alone, Table S1e: Conversion to styrene carbonate and diol by-product using amino acids alone, Figure S1: Variation in kelp catalyst loading in the synthesis of styrene carbonate, Table S2: Effect of CO_2_ pressure on conversion to styrene carbonate using kelp D < 125 nm as a catalyst, Figure S2: Conversion to styrene carbonate over time using kelp D catalyst and larger reaction scale of 20 mmols in a single Parr reactor, Table S3: Calculation of water content from TGIR analysis of unused kelp seaweeds B–D < 125, Table S4: % Organic vs inorganic content for kelp seaweeds B, C and D, Figure S3: TGIR Analysis of kelp B (Red), C (Blue) and D (Green). TG used to assess residual moisture content of the dried seaweeds, Figure S4: IR over time to measure water loss. TGIR of kelp B (left), kelp C (middle) and kelp D (right), Figure S5: ^1^H NMR of conversion to styrene carbonate using Kelp B 300-500 nm particle size as shown in table 3 along with an example carbonate conversion calculation, Figure S6: High resolution M/S of reaction mixture-conversion to styrene carbonate using kelp D < 125 nm particle size as shown in table 3, Figure S7: M/S of Peak 1—Benzaldehyde, Figure S8: M/S of Peak 2—Styrene Oxide, Figure S9: M/S of Peak 3—1-Phenyl-2-chloroethanol, Figure S10: M/S of Peak 4—1-Phenyl-1,2-ethanediol, Figure S11: M/S of Peak 5—Styrene carbonate, Figure S12: High resolution M/S of reaction mixture showing phenylacetaldehyde, 1-phenyl-1,2-ethanediol and styrene carbonate, Figure 13: ICP-MS raw data, Method S1: Detailed HPLC method for amino acid analysis.
