# Supplementary material for: Laminaria digitata and Palmaria palmata Seaweeds as Natural Source of Catalysts for the Cycloaddition of CO2 to Epoxides
Source: Molecules. 2019 Jan 12;24(2):269. doi: 10.3390/molecules24020269 (PMC6359002; doi:10.3390/molecules24020269)
Supplement: Supplementary file 1 [file molecules-24-00269-s001.pdf]

Supplementary Information

Laminaria digitata and *Palmaria palmata* Seaweeds as Natural Source of Catalysts for the Cycloaddition of CO_2 to Epoxides

James W. Comerford*, Thomas Gray, Yann Lie, Duncan J. Macquarrie, Michael North

and Alessandro Pellis

Contents

Table / Figure	Description	Page
Table S1a	Conversions to styrene carbonate and diol by-product using metal halides and histidine co-catalyst	2
Table S1b	Conversion to styrene carbonate and diol by-product using metal halides lysine co-catalyst	2
Table S1c	Conversion to styrene carbonate and diol by-product using metal halides glycine co-catalyst	2
Table S1d	Conversion to styrene carbonate and diol by-product using metal halides alone	2
Table S1e	Conversion to styrene carbonate and diol by-product using amino acids alone	2
Figure S1	Variation in Kelp catalyst loading in the synthesis of styrene carbonate	3
Table S2	Effect of CO_2 pressure on conversion to styrene carbonate using Kelp D <125 nm as a catalyst	3
Figure S2	Conversion to styrene carbonate over time using Kelp D catalyst and larger reaction scale of 20 mmols in a single Parr reactor.	3
Table S3	Calculation of water content from TGIR analysis of unused Kelp seaweeds B-D <125	4
Table S4	% Organic vs inorganic content for Kelp seaweeds B, C and D	4
Figure S3	TGIR Analysis of Kelp B (Red), C (Blue) and D (Green). TG used to assess residual moisture content of the dried seaweeds	5
Figure S4	IR over time to measure water loss. TGIR of Kelp B (left), Kelp C (middle) and Kelp D (right)	6
Figure S5	^{1}H NMR of conversion to styrene carbonate using Kelp B 300-500 nm particle size as shown in table 3 along with an example carbonate conversion calculation	7
Figure S6	GC/MS Chromatogram of conversion to styrene carbonate using Kelp D <125 nm particle size as shown in table 3.	8
Figure S7	M/S of Peak 1 - Benzaldehyde	9
Figure S8	M/S of Peak 2 – Styrene Oxide	10
Figure S9	M/S of Peak 3 – 1-Phenyl-2-chloroethanol	11
Figure S10	M/S of Peak 4 – 1-Phenyl-1,2-ethanediol	12
Figure S11	M/S of Peak 5 – Styrene carbonate	13
Figure S12	High resolution M/S of reaction mixture showing phenylacetaldehyde, 1-phenyl-1,2-ethanediol and styrene carbonate	14
Figure S13	ICP-MS raw data	15-22
Method S1	Detailed HPLC method for amino acid analysis	23-24

Table S1a) Conversion to styrene carbonate and diol by-product using metal halides and histidine co-catalyst

Conversion %	Metal Halide						
	KI	KBr	KCl	NaI	NaBr	NaCl	$CaCl_2$
**Carbonate 2a**	75.2	76.3	61.7	49.8	67.2	77.5	80.0
**Diol 3a**	0	23.7	21.0	3.9	32.8	8.3	17.6
**Epoxide 1a**	24.8	0	17.3	18.6	0	42.1	2.4

Table S1b) Conversion to styrene carbonate and diol by-product using metal halides lysine co-catalyst

Conversion %	Metal Halide						
	KI	KBr	KCl	NaI	NaBr	NaCl	$CaCl_2$
**Carbonate 2a**	63.9	44.1	8.0	56.2	58.8	12.1	18.7
**Diol 3a**	14.6	3.5	5.1	16.8	3.5	6.5	13.9
**Epoxide 1a**	21.5	52.4	86.9	27.0	37.7	81.4	67.4

Table S1c) Conversion to styrene carbonate and diol by-product using metal halides glycine co-catalyst

Conversion %	Metal Halide						
	KI	KBr	KCl	NaI	NaBr	NaCl	$CaCl_2$
**Carbonate 2a**	79.5	10.2	0.8	97.1	41.2	0	11.8
**Diol 3a**	11.5	2.4	1.6	2.9	3.7	1.7	9.1
**Epoxide 1a**	9.0	87.4	97.6	0	55.1	98.3	79.1

Table S1d) Conversion to styrene carbonate and diol by-product using metal halides alone

Conversion %	Metal Halide						
	KI	KBr	KCl	NaI	NaBr	NaCl	$CaCl_2$
**Carbonate 2a**	100	0.9	1.0	23.1	2.8	1.0	0
**Diol 3a**	0	4.7	2.9	1.5	3.7	4.7	13.8
**Epoxide 1a**	0	94.3	96.2	75.4	93.5	94.3	86.2

Table S1e) Conversion to styrene carbonate and diol by-product using amino acids alone

Conversion %	Amino acid		
	Histidine	Lysine	Glycine
**Carbonate 2a**	39.1	9.7	0
**Diol 3a**	15.2	9.4	0
**Epoxide 1a**	45.7	80.9	100

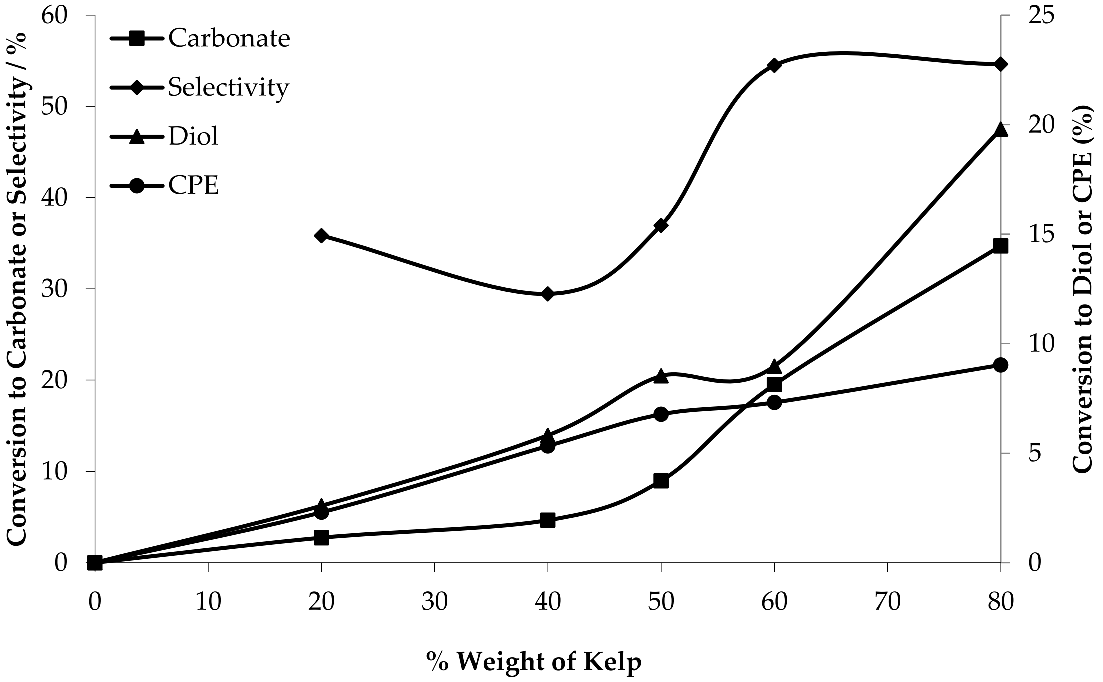

Figure S1. Variation in Kelp catalyst loading in the synthesis of styrene carbonate

Table S2. Effect of CO_2 pressure on conversion to styrene carbonate using Kelp D <125 nm as a catalyst

Pressure / bar	Carbonate / %	Diol / %	Epoxide / %	CPE / %	Selectivity / %
10	26.5	26.1	33.7	8.6	43.3
20	31.1	29.7	28.1	11.1	43.3
30	36.1	23.0	37.8	3.1	58.0
40	39.4	35.8	13.3	11.5	45.5

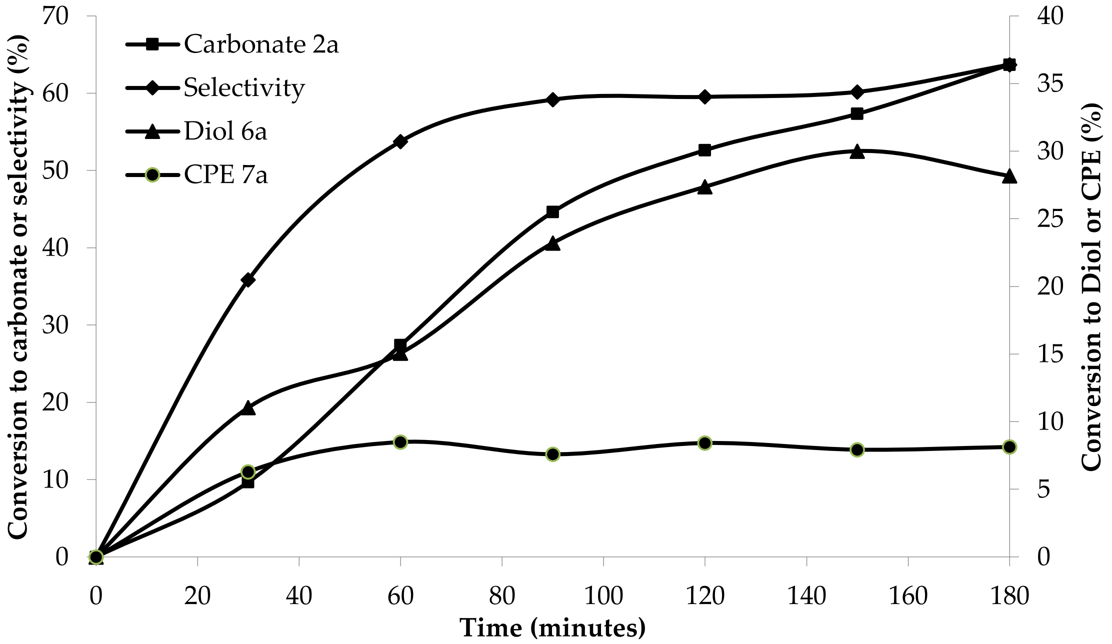

Figure S2. Conversion to styrene carbonate over time using Kelp D catalyst and larger reaction scale of 20 mmols in a single Parr reactor.

Table S3. Calculation of water content from TGIR analysis of unused Kelp seaweeds B-D <125

Kelp	Water content TGIR (% wt)	Particle Size (μm)	Conversion to Diol 6a (%)	Diol (mmol)	H_2O in Kelp Sample (mmol)	H_2O converted to diol (%)
B (18/09/17)	8.2	< 125	26.2	1.31	2.17	60.4
C (15/11/17)	8.5	< 125	27.4	1.37	2.25	60.9
D (20/11/17)	8.4	< 125	35.2	1.76	2.22	79.2

Table S4. % organic vs inorganic content for Kelp seaweeds B, C and D

Kelp		% Weight of residual inorganics	% Weight organic material lost
B	Unused	24.0	76.0
	Used	33.0	67.0
C	Unused	22.1	77.9
	Used	32.7	67.3
D	Unused	23.8	76.2
	Used	32.6	67.4

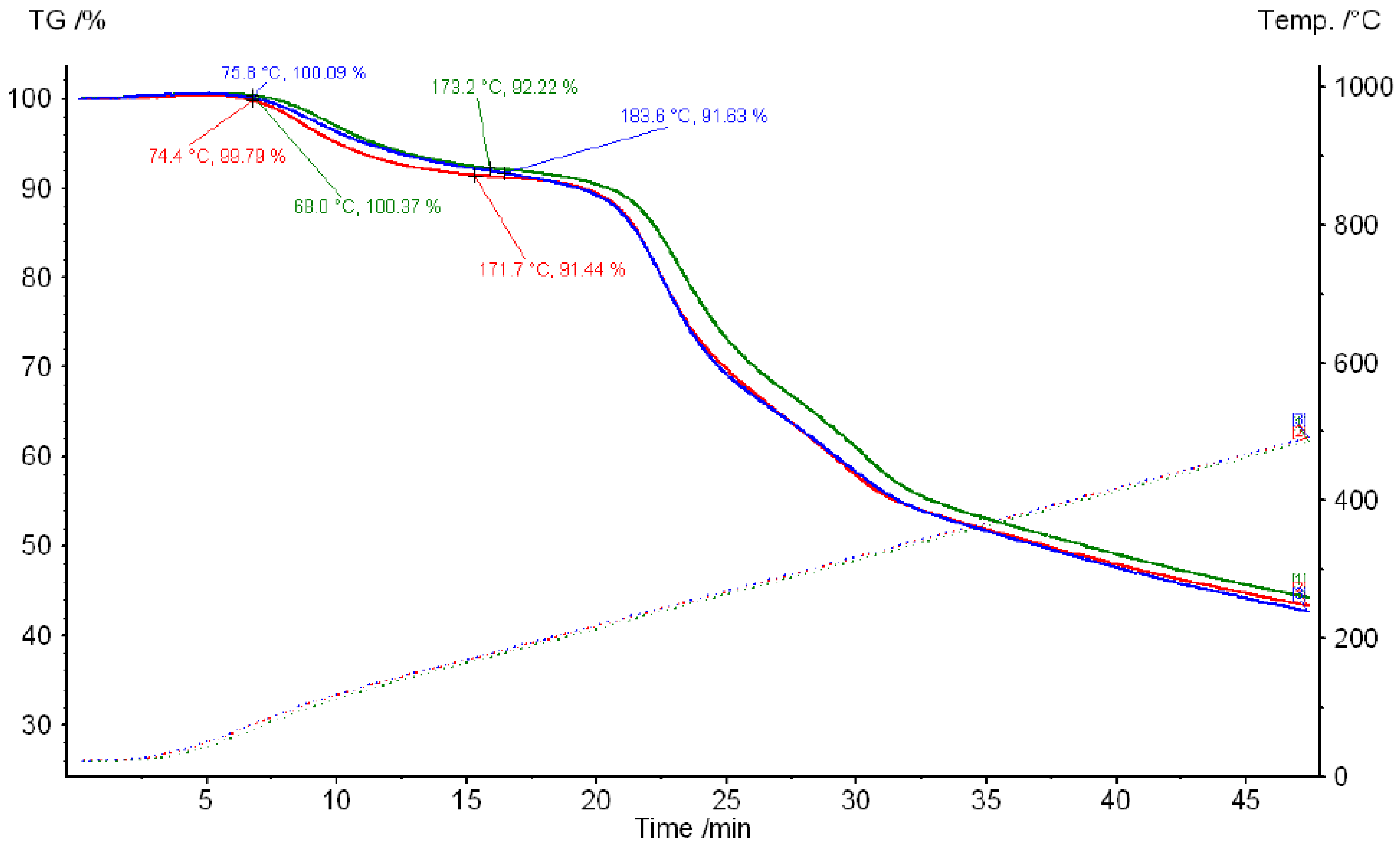

Figure S3. TGIR Analysis of Kelp B (Green), C (Blue) and D (Red). TG used to assess residual moisture content of the dried seaweeds

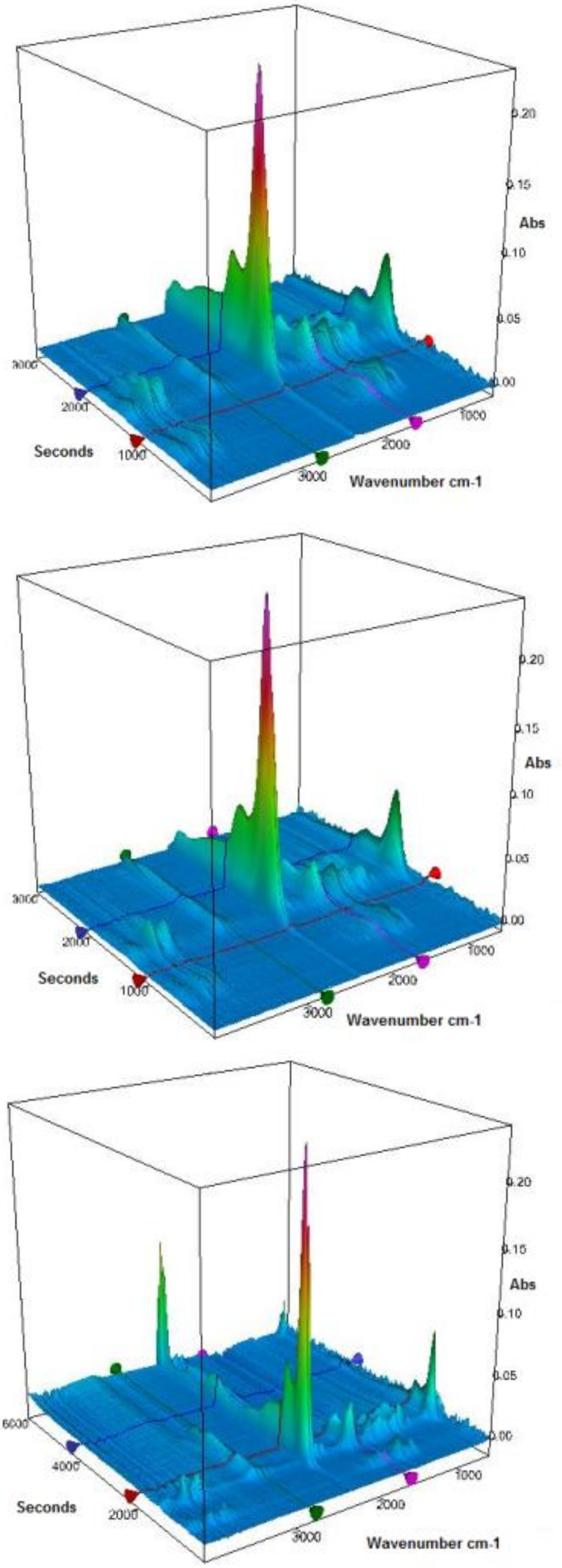

Figure S4. TGIR to measure residual water in Kelp B (top), Kelp C (middle) and Kelp D (bottom)

$$\mathit{Conversion\ to\ carbonate}\ (19.9) = 100\ \times \frac{H1\ (13.78)}{H1\ (13.78) + H2\ (7.17) + H3(16.16) + H5(30.11) + H6(0.38) + H7(1.55)}$$

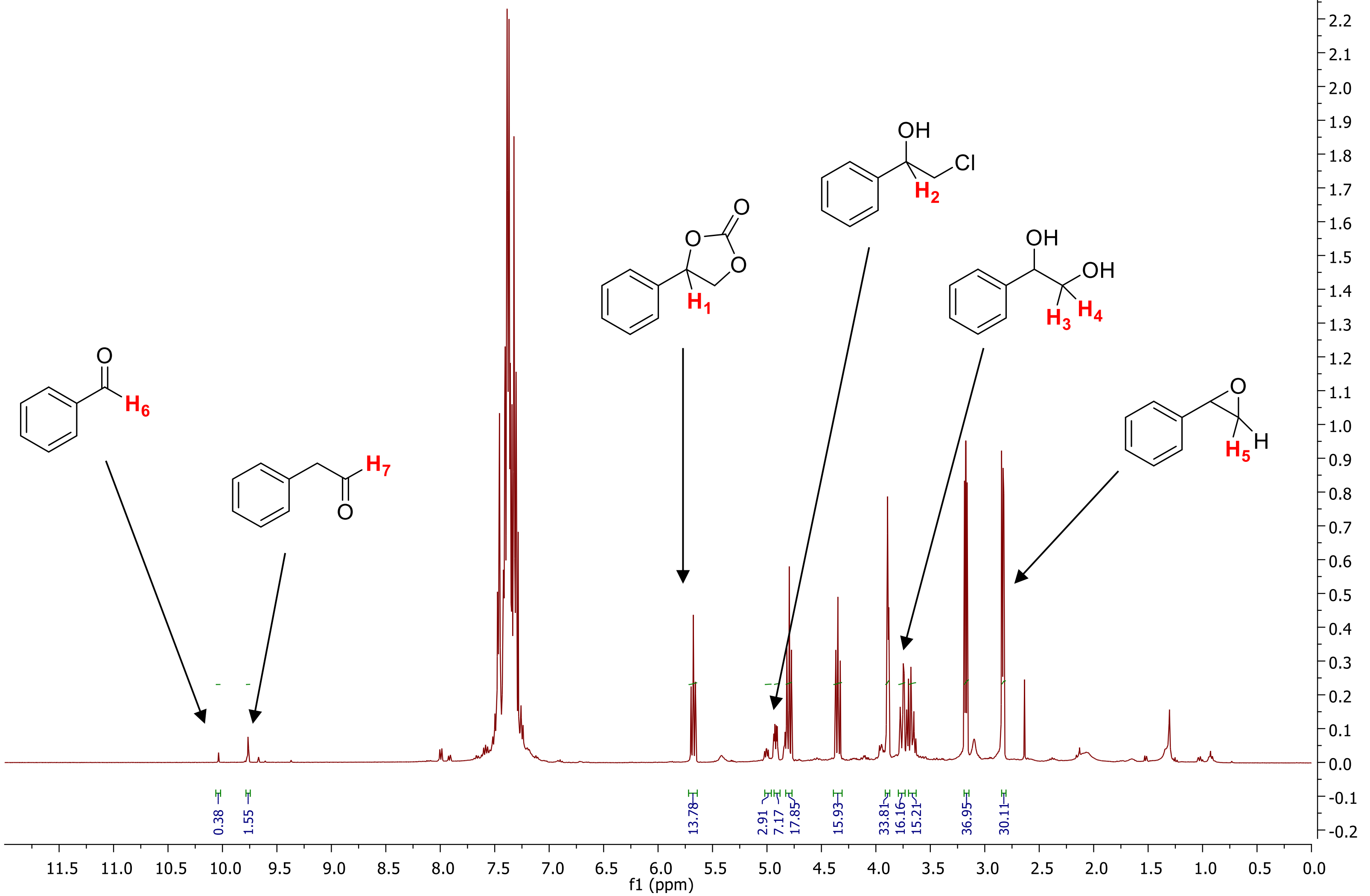

Figure S5. ^{1}H NMR of conversion to styrene carbonate using Kelp B 300-500 nm particle size as shown in table 3.

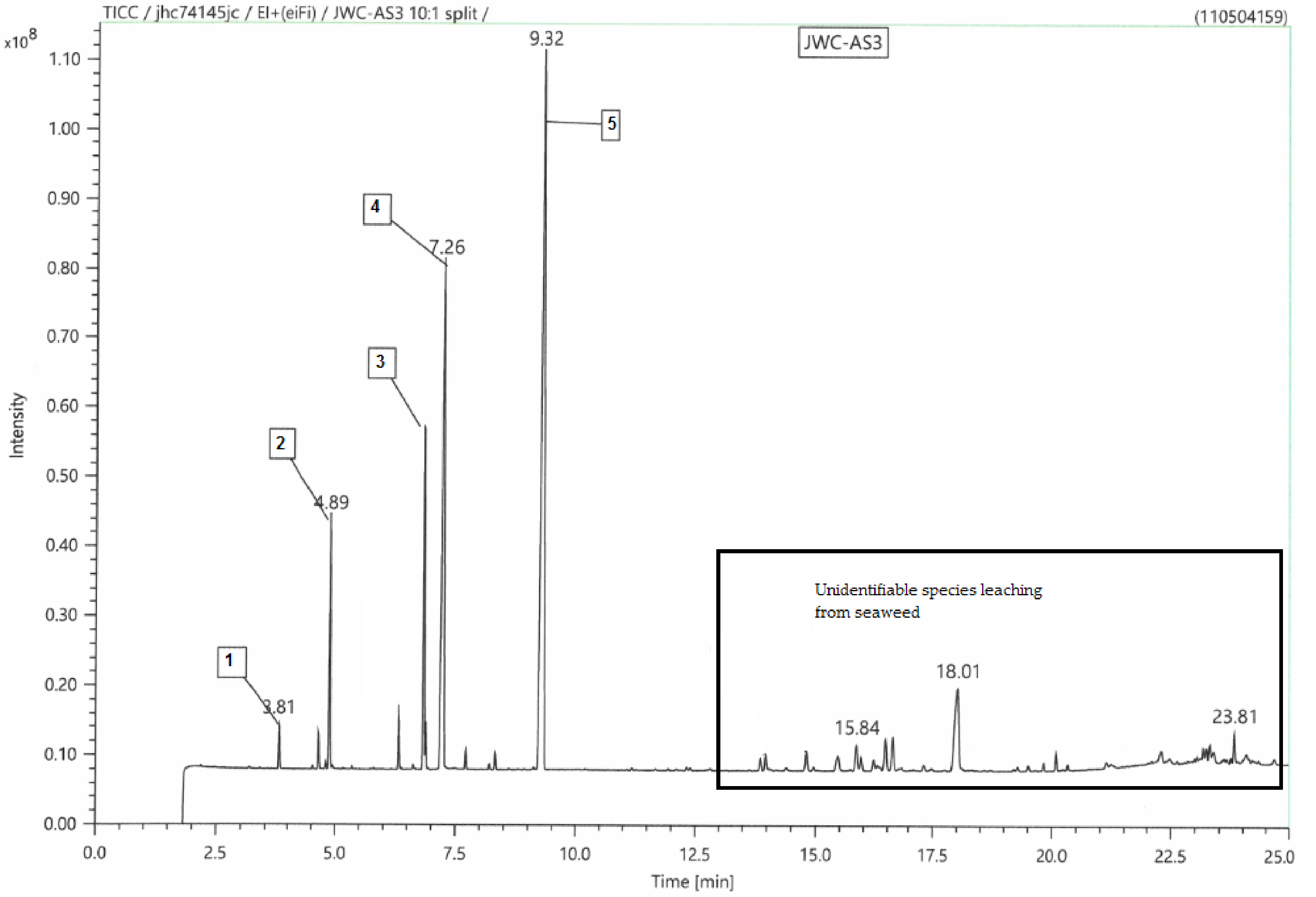

Figure S6. GC/MS Chromatogram of conversion to styrene carbonate using Kelp D <125 nm particle size as shown in table 3.

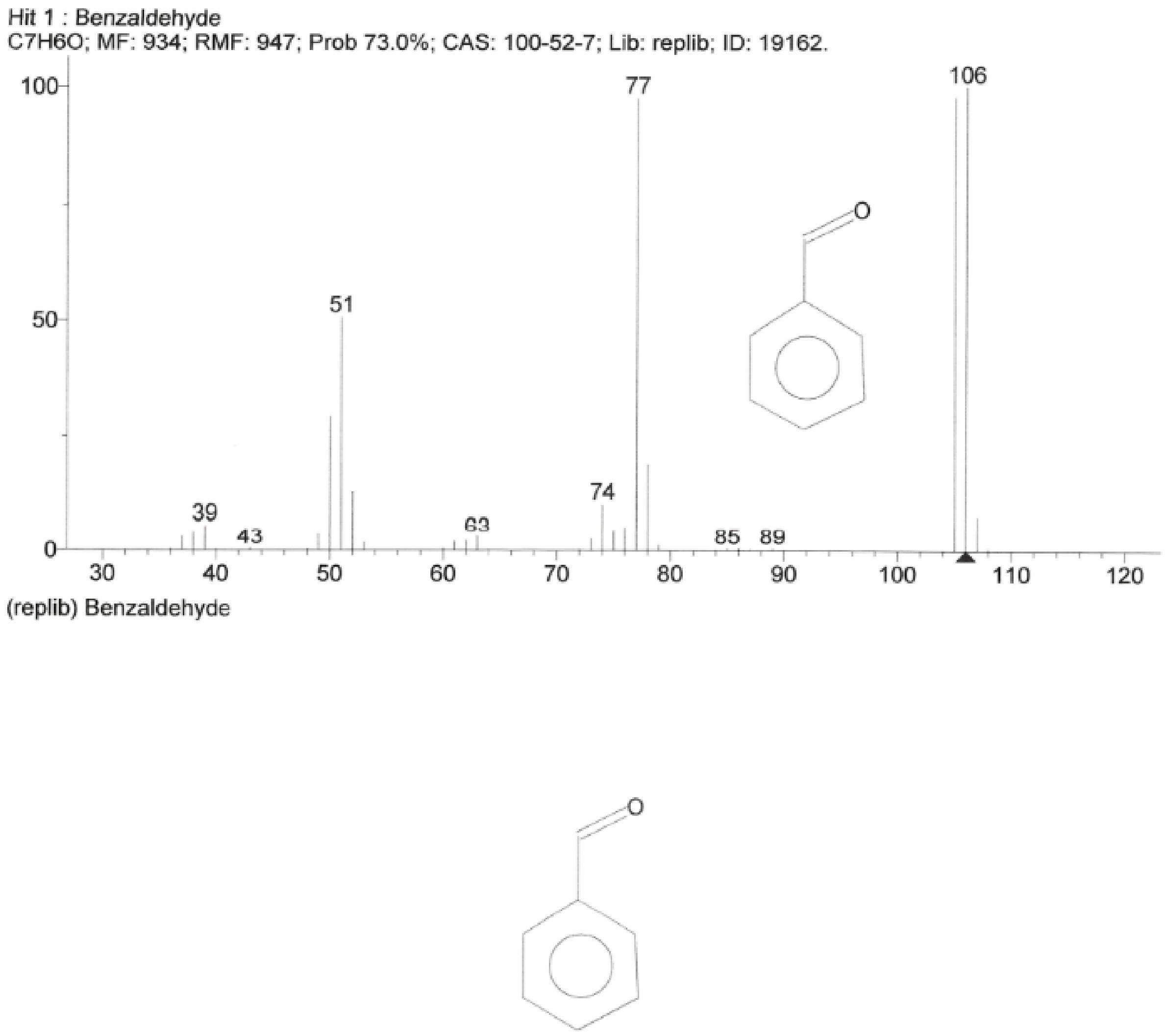

Figure S7. M/S of Peak 1 - Benzaldehyde

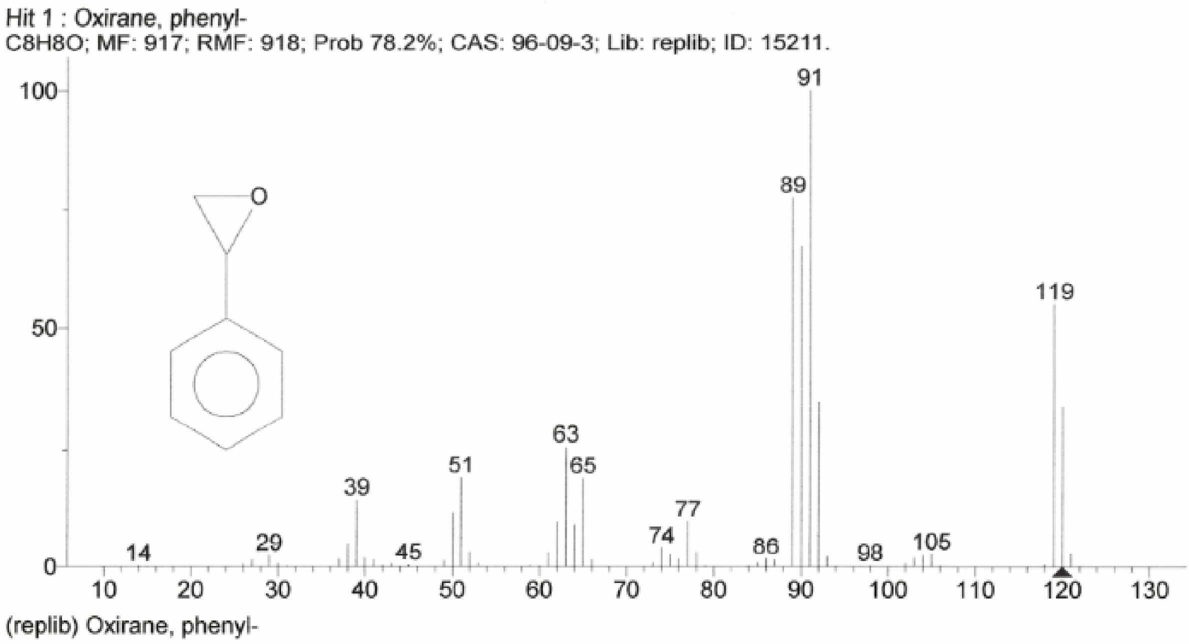

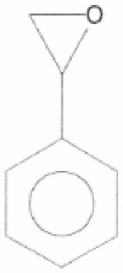

Figure S8. M/S of Peak 1 – Styrene Oxide

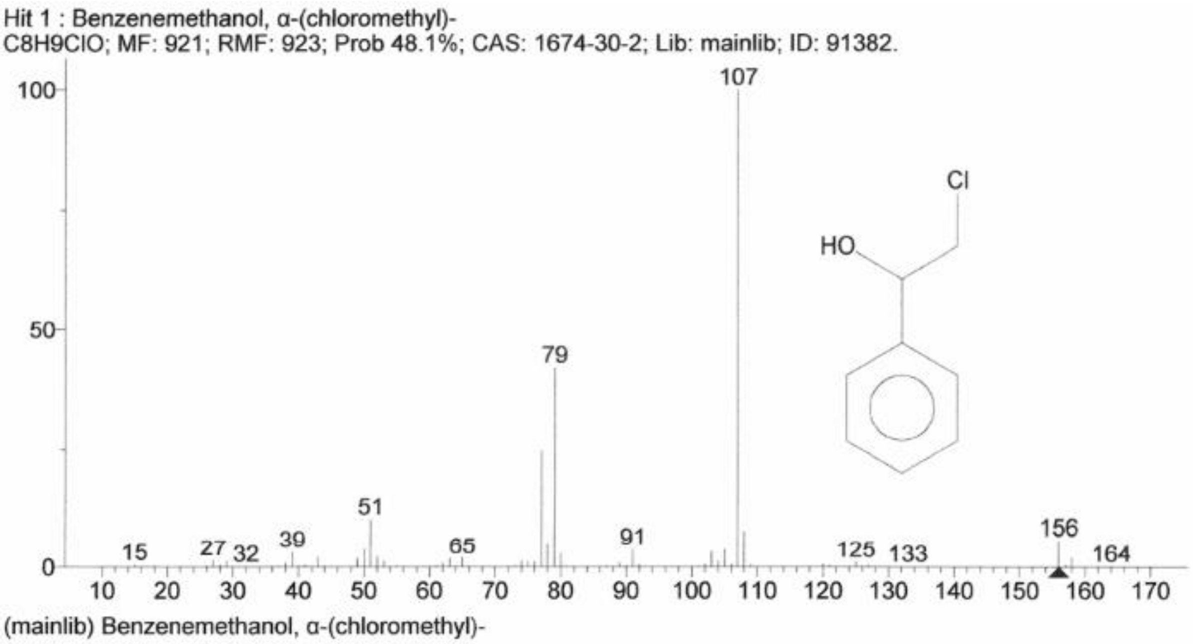

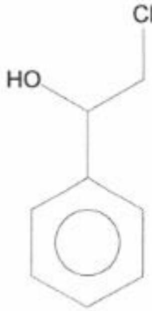

Figure S9. M/S of Peak 3 – 1-Phenyl-2-chloroethanol

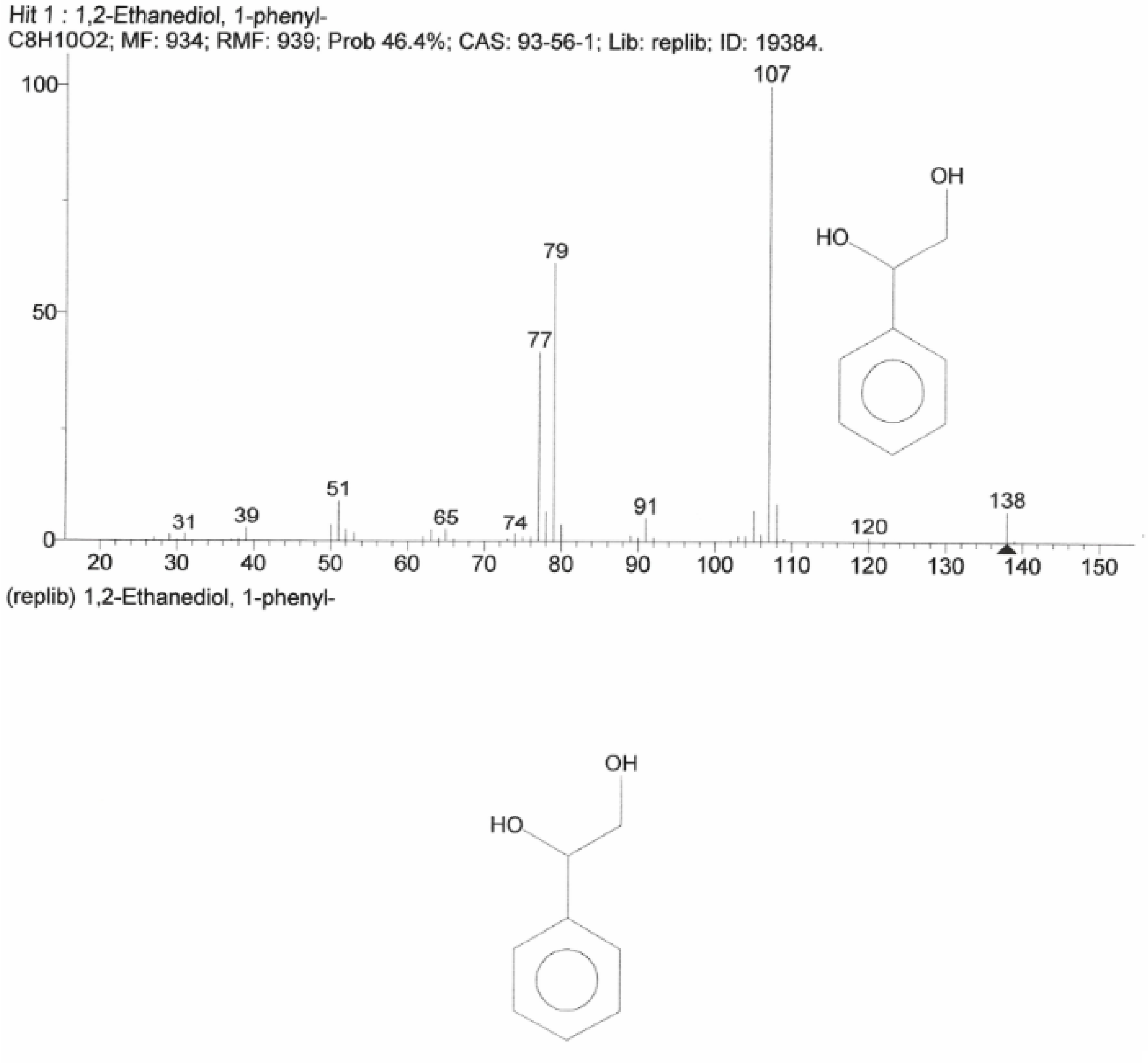

Figure S10. M/S of Peak 4 – 1-Phenyl-1,2-ethanediol

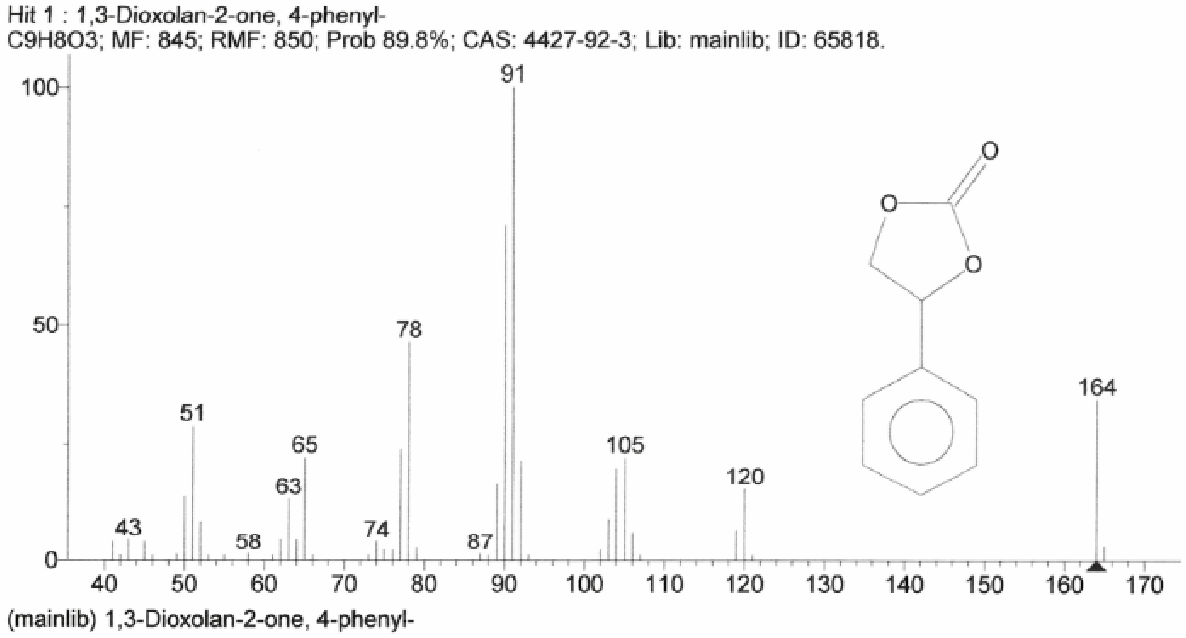

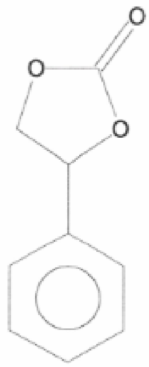

Figure S11. M/S of Peak 5 – Styrene carbonate

York - Chemistry - Mass Spectrometry Service Report *JWC AS3*

Analysis Information

Analysis Filename	jhc74122jc_P1-E-2_01_9513.d	Acquisition Date	06/12/2018 12:53:27
Method	ESI_low mass_2c1s.m	Instrument	compact
Submission Name	jhc74122jc	ESI	Positive

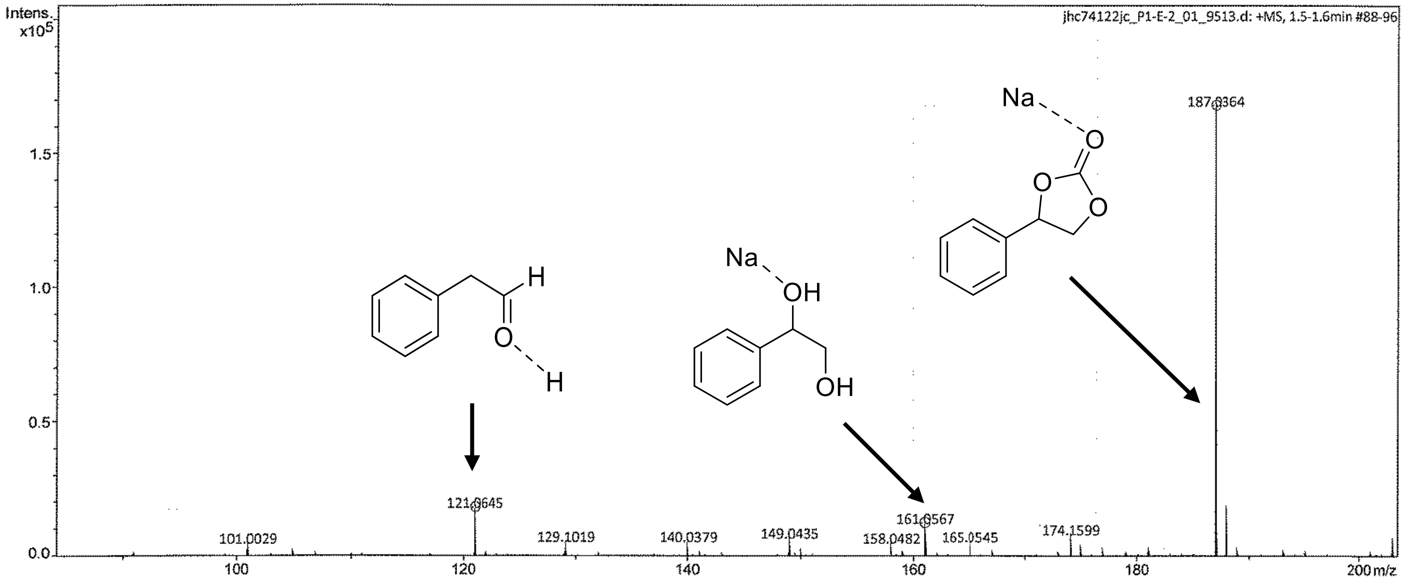

Meas. m/z	#	Ion Formula	m/z	err [ppm]	err [mDa]	mSigma	Mean err [ppm]
121.0645	1	C8H9O	121.0648	2.5	0.3	4.3	1.0
161.0567	1	C8H10NaO2	161.0573	4.0	0.6	4.6	3.3
187.0364	1	C9H8NaO3	187.0366	0.7	0.1	2.7	-0.3

Figure S12. High resolution M/S of reaction mixture showing phenylacetaldehyde, 1-phenyl-1,2-ethanediol and styrene carbonate

Figure S13. ICP-MS raw data

**Sample ID**	**Analyte Name**	**Int (Corr)**	**RSD (Corr Int)**	**SD (Corr Int)**	**Conc (mg l-1)**	**RSD (%)**	**SD (mg l-1)**	**Corr Coef**		
Blank	Al 396.153	56913.40945	0.78103	444.51304						
0.01 ppm M4	Al 396.153	1559.31457	86.22568	1344.52961						
0.05 ppm M4	Al 396.153	3274.73052	48.09511	1574.98518						
0.1 ppm M4	Al 396.153	6194.72994	10.58408	655.65499						
0.5 ppm M4	Al 396.153	34537.18784	1.04251	360.05218					**mg metal / 100ml**	**mg Metal / g seaweed**
Kelp D	Al 396.153	10402.29670	10.32365	1073.89719	0.15077	10.37040	0.01564	0.99924	0.01508	0.36138
Kelp C	Al 396.153	8493.08945	7.05515	599.20011	0.12297	7.09432	0.00872	0.99924	0.01230	0.27176
Kelp B	Al 396.153	6412.83655	15.57553	998.83339	0.09268	15.69025	0.01454	0.99924	0.00927	0.22099
M6 x 100	Al 396.153	82684.57059	5.89266	4872.32164	1.20316	5.89600	0.07094	0.99924		
SLRS-4	Al 396.153	11190.60560	15.03809	1682.85377	0.16225	15.10137	0.02450	0.99924		
**Sample ID**	**Analyte Name**	**Int (Corr)**	**RSD (Corr Int)**	**SD (Corr Int)**	**Conc (mg l-1)**	**RSD (%)**	**SD (mg l-1)**	**Corr Coef**		
Blank	Ca 317.933	2967.10570	1.50992	44.80098						
0.01 ppm M4	Ca 317.933	870.59020	5.68091	49.45744						
0.05 ppm M4	Ca 317.933	3944.34839	4.36552	172.19138						
0.1 ppm M4	Ca 317.933	7585.59997	1.81528	137.70015						
0.5 ppm M4	Ca 317.933	35112.42619	1.38776	487.27515						

1 ppm M4	Ca 317.933	71087.96518	1.13220	804.85508						
5 ppm M4	Ca 317.933	362272.60650	0.22413	811.97229						
10 ppm M4	Ca 317.933	685340.26360	0.77909	5339.42207					mg metal / 100ml	mg Metal / g seaweed
Kelp D	Ca 317.933	384662.12620	1.08011	4154.76088	5.54049	1.08550	0.06014	0.99967	0.55405	13.28017
Kelp C	Ca 317.933	454356.86490	0.52874	2402.36087	6.54935	0.53097	0.03478	0.99967	0.65494	14.47370
Kelp B	Ca 317.933	499804.70170	1.40063	7000.40822	7.20723	1.40601	0.10133	0.99967	0.72072	17.18462
M6 x 100	Ca 317.933	7176085.0480	1.94243	139390.2383	103.84971	1.94295	2.01774	0.99967		
SLRS-4	Ca 317.933	468697.19730	1.63826	7678.48136	6.75693	1.64497	0.11115	0.99967		
**Sample ID**	**Analyte Name**	**Int (Corr)**	**RSD (Corr Int)**	**SD (Corr Int)**	**Conc (mg l^{-1})**	**RSD (%)**	**SD (mg l^{-1})**	**Corr Coef**		
Blank	Fe 239.562	825.59447	7.20401	59.47593						
0.01 ppm M4	Fe 239.562	68.20726	15.97451	10.89577						
0.05 ppm M4	Fe 239.562	1409.20400	0.91541	12.89995						
0.1 ppm M4	Fe 239.562	3121.33455	1.56229	48.76423						
0.5 ppm M4	Fe 239.562	16491.41643	0.82424	135.92827					mg metal / 100ml	mg Metal / g seaweed
Kelp D	Fe 239.562	2746.88444	3.48247	95.65939	0.08783	3.26968	0.00287	0.99988	0.00878	0.21053
Kelp C	Fe 239.562	1373.45272	56.35865	774.05938	0.04660	49.86780	0.02324	0.99988	0.00466	0.10298
Kelp B	Fe 239.562	1337.03477	1.66993	22.32755	0.04551	1.47298	0.00067	0.99988	0.00455	0.10851
M6 x 100	Fe 239.562	321508.12650	2.40486	7731.81342	9.65763	2.40352	0.23212	0.99988		
SLRS-4	Fe 239.562	3758.87094	1.49273	56.10963	0.11822	1.42496	0.00168	0.99988		

Sample ID	Analyte Name	Int (Corr)	RSD (Corr Int)	SD (Corr Int)	Conc (mg l-1)	RSD (%)	SD (mg l-1)	Corr Coef		
Blank	K 766.490	39411.77750	0.60793	239.59536						
0.01 ppm M4	K 766.490	834.34351	79.27820	661.45255						
0.05 ppm M4	K 766.490	7799.79558	13.91678	1085.48006						
0.1 ppm M4	K 766.490	18395.20590	3.37716	621.23569						
0.5 ppm M4	K 766.490	121343.38290	1.18965	1443.56055						
1 ppm M4	K 766.490	269818.74600	1.24409	3356.79652						
5 ppm M4	K 766.490	1553987.3690	0.12814	1991.25890						
10 ppm M4	K 766.490	3447665.6020	0.74695	25752.28945					mg metal / 100ml	mg Metal / g seaweed
Kelp D	K 766.490	3592594.1290	1.92802	69265.82656	10.61271	1.90984	0.20269	0.99890	1.06127	25.43794
Kelp C	K 766.490	2202196.2930	0.45530	10026.70899	6.54413	0.44834	0.02934	0.99890	0.65441	14.46216
Kelp B	K 766.490	2166705.1830	3.73589	80945.68037	6.44027	3.67785	0.23686	0.99890	0.64403	15.35592
M6 x 100	K 766.490	321884.13760	3.06666	9871.08468	1.04195	2.77218	0.02888	0.99890		
SLRS-4	K 766.490	165143.70500	4.01746	6634.58766	0.58330	3.32833	0.01941	0.99890		
**Sample ID**	**Analyte Name**	**Int (Corr)**	**RSD (Corr Int)**	**SD (Corr Int)**	**Conc (mg l-1)**	**RSD (%)**	**SD (mg l-1)**	**Corr Coef**		
Blank	Li 670.784	34214.85071	1.65450	566.08502						
0.01 ppm M4	Li 670.784	76763.87060	5.79995	4452.26712						
0.05	Li 670.784	356810.23520	1.56885	5597.82175						

ppm M4										
0.1 ppm M4	Li 670.784	721239.65080	1.66340	11997.11059						
0.5 ppm M4	Li 670.784	3970472.1170	3.4764	138030.8475					mg metal / 100ml	mg Metal / g seaweed
Kelp D	Li 670.784	50824.94876	2.77341	1409.58426	0.00984	1.79677	0.00018	0.99982	0.00098	0.02358
Kelp C	Li 670.784	37524.70326	2.87641	1079.36306	0.00817	1.65672	0.00014	0.99982	0.00082	0.01805
Kelp B	Li 670.784	34432.66517	5.05723	1741.33784	0.00778	2.80596	0.00022	0.99982	0.00078	0.01855
M6 x 100	Li 670.784	10918312.900	1.5607	170398.8734	1.37255	1.55673	0.02137	0.99982		
SLRS-4	Li 670.784	2040.30678	157.87215	3221.07619	0.00372	10.85775	0.00040	0.99982		
**Sample ID**	**Analyte Name**	**Int (Corr)**	**RSD (Corr Int)**	**SD (Corr Int)**	**Conc (mg l^{-1})**	**RSD (%)**	**SD (mg l^{-1})**	**Corr Coef**		
Blank	Mg 279.077	947.55371	2.48345	23.53202						
0.01 ppm M4	Mg 279.077	103.97117	11.33641	11.78660						
0.05 ppm M4	Mg 279.077	460.50814	3.34628	15.40989						
0.1 ppm M4	Mg 279.077	883.57931	8.55461	75.58680						
0.5 ppm M4	Mg 279.077	4601.51856	0.27202	12.51700						
1 ppm M4	Mg 279.077	9255.24152	0.85013	78.68118						
5 ppm M4	Mg 279.077	43245.81425	0.54501	235.69615						
10 ppm M4	Mg 279.077	85647.67687	0.35866	307.18094					mg metal / 100ml	mg Metal / g seaweed

Kelp D	Mg 279.077	30641.36225	1.37547	421.46280	3.55591	1.38418	0.04922	0.99996	0.35559	8.52328
Kelp C	Mg 279.077	40544.61367	1.72902	701.02425	4.71246	1.73728	0.08187	0.99996	0.47125	10.41427
Kelp B	Mg 279.077	37131.34058	0.88000	326.75466	4.31384	0.88459	0.03816	0.99996	0.43138	10.28574
M6 x 100	Mg 279.077	8689.81949	0.45480	39.52141	0.99232	0.46512	0.00462	0.99996		
SLRS-4	Mg 279.077	14604.98794	1.33611	195.13831	1.68312	1.35397	0.02279	0.99996		
**Sample ID**	**Analyte Name**	**Int (Corr)**	**RSD (Corr Int)**	**SD (Corr Int)**	**Conc (mg l^{-1})**	**RSD (%)**	**SD (mg l^{-1})**	**Corr Coef**		
Blank	Na 589.592	95057.20193	1.88720	1793.91672						
0.01 ppm M4	Na 589.592	4077.88123	101.12038	4123.56888						
0.05 ppm M4	Na 589.592	19024.14446	25.22180	4798.23091						
0.1 ppm M4	Na 589.592	42461.04606	5.16732	2194.09761						
0.5 ppm M4	Na 589.592	249823.22100	3.09198	7724.47814						
1 ppm M4	Na 589.592	522859.85670	1.36570	7140.71527						
5 ppm M4	Na 589.592	3146361.088	0.72796	22904.11598						
10 ppm M4	Na 589.592	6824688.653	0.51667	35261.12651					mg metal / 100ml	mg Metal / g seaweed
Kelp D	Na 589.592	11639595.610	0.39904	46446.57274	17.24786	0.39694	0.06846	0.99923	1.72479	41.34196
Kelp C	Na 589.592	13419144.840	0.14953	20065.51438	19.87096	0.14885	0.02958	0.99923	1.98710	43.91372
Kelp B	Na 589.592	13779426.880	0.67480	92984.01208	20.40202	0.67180	0.13706	0.99923	2.04020	48.64573
M6 x 100	Na 589.592	642577.239	4.45447	28623.42983	1.03803	4.06457	0.04219	0.99923		
SLRS-4	Na 589.592	1117909.105	1.27592	14263.64454	1.73868	1.20925	0.02102	0.99923		

Sample ID	Analyte Name	Int (Corr)	RSD (Corr Int)	SD (Corr Int)	Conc (mg l^{-1})	RSD (%)	SD (mg l^{-1})	Corr Coef		
Blank	Zn 202.548	68.40420	11.52839	7.88590						
0.01 ppm M4	Zn 202.548	170.52294	5.87456	10.01747						
0.05 ppm M4	Zn 202.548	905.69475	1.06340	9.63114						
0.1 ppm M4	Zn 202.548	1767.78131	2.19623	38.82454						
0.5 ppm M4	Zn 202.548	9086.78076	0.60040	54.55745					mg metal / 100ml	mg Metal / g seaweed
Kelp D	Zn 202.548	1953.92914	0.74491	14.55509	0.10824	0.73918	0.00080	0.99999	0.01082	0.25945
Kelp C	Zn 202.548	2703.58040	15.50297	419.13530	0.14945	15.41652	0.02304	0.99999	0.01494	0.33027
Kelp B	Zn 202.548	5078.83806	1.03871	52.75454	0.28002	1.03562	0.00290	0.99999	0.02800	0.66766
M6 x 100	Zn 202.548	165916.34310	1.66175	2757.11030	9.12123	1.66160	0.15156	0.99999		
SLRS-4	Zn 202.548	302.28066	7.89095	23.85281	0.01745	7.51409	0.00131	0.99999		
**Sample ID**	**Analyte Name**	**Int (Corr)**	**RSD (Corr Int)**	**SD (Corr Int)**	**Conc (mg l^{-1})**	**RSD (%)**	**SD (mg l^{-1})**	**Corr Coef**		
Blank	Sr 460.733	448.46187	29.57393	132.62778						
0.01 ppm M4	Sr 460.733	4681.59424	5.69415	266.57699						
0.05 ppm M4	Sr 460.733	23046.01900	4.43156	1021.29870						
0.1 ppm M4	Sr 460.733	46258.34326	0.90296	417.69295						
0.5 ppm M4	Sr 460.733	240906.09410	2.95058	7108.12851					mg metal / 100ml	mg Metal / g seaweed

Kelp D	Sr 460.733	225885.40580	2.26485	5115.95983	0.46946	2.25741	0.01060	0.99997	0.04695	1.12526
Kelp C	Sr 460.733	255123.45470	0.57645	1470.64704	0.53003	0.57477	0.00305	0.99997	0.05300	1.17133
Kelp B	Sr 460.733	274155.88850	4.05199	11108.76795	0.56945	4.04102	0.02301	0.99997	0.05695	1.35777
M6 x 100	Sr 460.733	571067.40570	3.19600	18251.33006	1.18450	3.19184	0.03781	0.99997		
SLRS-4	Sr 460.733	16404.34785	3.74642	614.57564	0.03552	3.58387	0.00127	0.99997		
**Sample ID**	**Analyte Name**	**Int (Corr)**	**RSD (Corr Int)**	**SD (Corr Int)**	**Conc (mg l^{-1})**	**RSD (%)**	**SD (mg l^{-1})**	**Corr Coef**		
Blank	Cu 324.752	7581.41265	1.39837	106.01653						
0.01 ppm M4	Cu 324.752	1852.48140	11.17210	206.96108						
0.05 ppm M4	Cu 324.752	11822.95445	5.27419	623.56545						
0.1 ppm M4	Cu 324.752	23795.80573	0.26927	64.07606						
0.5 ppm M4	Cu 324.752	123464.46870	0.92309	1139.68702					mg metal / 100ml	mg Metal / g seaweed
Kelp D	Cu 324.752	12070.18814	2.25050	271.63975	0.05081	2.15790	0.00110	0.99998	0.00508	0.12178
Kelp C	Cu 324.752	9495.19873	1.64382	156.08416	0.04041	1.55879	0.00063	0.99998	0.00404	0.08931
Kelp B	Cu 324.752	13418.59768	1.43810	192.97286	0.05625	1.38465	0.00078	0.99998	0.00562	0.13412
M6 x 100	Cu 324.752	249633.41230	2.48710	6208.64271	1.00963	2.48195	0.02506	0.99998		
SLRS-4	Cu 324.752	-716.00074	13.43241	96.17614	-0.00080	48.56552	0.00039	0.99998		
**Sample ID**	**Analyte Name**	**Int (Corr)**	**RSD (Corr Int)**	**SD (Corr Int)**	**Conc (mg l^{-1})**	**RSD (%)**	**SD (mg l^{-1})**	**Corr Coef**		
Blank	Ba 233.527	129.40965	7.43261	9.61852						
0.01 ppm M4	Ba 233.527	706.23876	2.23855	15.80951						
0.05 ppm M4	Ba 233.527	3548.79451	1.35061	47.93030						

0.1 ppm M4	Ba 233.527	7124.22001	1.12217	79.94551						
0.5 ppm M4	Ba 233.527	34344.81833	1.04266	358.09862					mg metal / 100ml	mg Metal / g seaweed
Kelp D	Ba 233.527	1996.79511	0.98931	19.75450	0.02777	1.03750	0.00029	0.99997	0.00278	0.06655
Kelp C	Ba 233.527	2964.02210	52.06314	1543.16300	0.04187	53.74497	0.02250	0.99997	0.00419	0.09253
Kelp B	Ba 233.527	1902.37748	1.19978	22.82429	0.02639	1.26127	0.00033	0.99997	0.00264	0.06292
M6 x 100	Ba 233.527	65934.11422	1.70437	1123.75821	0.96012	1.70677	0.01639	0.99997		
SLRS-4	Ba 233.527	954.11327	1.53323	14.62872	0.01256	1.69833	0.00021	0.99997		

Method S1. Detailed HPLC method for amino acid analysis

An Agilent HPLC infinity 1200 was used to perform the chromatography and a Poroshell 120 EC-C18 4.6 x 100 mm, 2.7 μm diameter. Temperature of the oven was set at 45 ◦C, a quaternary pump (G7111B) was used to pump eluents through the column with a pressure limit of 600 bar and a flow set at 0.4 mL/min. A DAD detector (G7115A) was set at wavelength 263 nm (FMOC) and 338 nm (OPA). Settings of the sampler (G7129A) were the following: draw speed-200 μL/min, eject speed-400 μL/min.

The following eluent gradient was used:

Time	A	B
min	%	%
12.00	80.0	20.0
13.00	65.0	35.0
17.00	60.0	40.0
19.00	55.0	45.0
21.40	44.7	55.3
25.00	40.0	60.0
28.00	30.0	70.0
29.20	25.0	75.0
30.00	15.0	85.0
32.00	10.0	90.0
34.00	5.0	95.0
36.00	0.0	100.0
37.00	40.0	60.0
40.00	80.0	20.0

Eluent A (2L) was prepared as follow: 2.839 g of Na_2HPO_4 and 4.024 g of Na_2B4O7 were dissolved in 2 L DI water and adjusted to pH 7.9 with conc. HCl (37%) and 1M HCl.

Eluent B consisted (1 L) in a MeOH: MeCN: H2O 20:60:20 solution.

Borate Buffer (BB) solution was made by dissolving 1.24 g of Boric Acid and 1.49 g of KCl in 50 mL DI water adjusted to pH 10.5 with NaOH ground pellets.

Ethanethiol solution (ETSH) consisted of 500 μL Ethanethiol, 20mL BB and 80 mL MeOH.

FMOC solution was prepared dissolving 25 mg of FMOC Chloride in 10 mL MeCN. OPA/ET was done dissolving 40 mg of OPA in 10 mL ET solution.

Diluent was prepared by mixing 48.5 mL of eluent A with 1.5 mL concentrated phosphoric acid.

Internal standard solution consisted (ISTDsol) in a 1.5 mM Norleucine solution in water.

The pre-column derivatisation was done in the HPLC needle by an automated procedure as follows:

Auto-sampler program for pre-column derivatisation

Function	Parameter
Draw	Draw 50.00 µL from location "P2-A1" with default speed using default offset
Draw	Draw 1.00 µL from air with default speed
Draw	Draw 2.00 µL from location "P2-A2" with default speed using default offset
Draw	Draw 2.00 µL from sample with default speed using default offset
Draw	Draw 12.00 µL from air with default speed
Mix	Mix 4.00 µL from air with default speed for 5 times
Wait	Wait 1 min
Eject	Eject 12.00 µL to seat with default speed
Wash	Wash needle in flushport for 5 s
Draw	Draw 2.00 µL from location "P2-A3" with default speed using default offset
Draw	Draw 12.00 µL from air with default speed
Mix	Mix 6.00 µL from air with default speed for 5 times
Wait	Wait 0.15 min
Eject	Eject 12.00 µL to seat with default speed
Wash	Wash needle in flushport for 5 s
Draw	Draw 4.00 µL from location "P2-A4" with default speed using default offset
Draw	Draw 12.00 µL from air with default speed
Mix	Mix 10.00 µL from air with default speed for 5 times
Eject	Eject 12.00 µL to seat with default speed
Inject	Inject

Hydrolysis of the microalgae samples was done as follow: 20 mg of algal powder in 25 mL 6M HCl with 1% (w/v phenol) were heated at 150 ◦C for 30 min in a CEM discover microwave. The resulting mixture was filtered and evaporated under reduce pressure. The resulting solid was re-suspended in 30 mL of suspension solution consisting of Water:MeOH:ISTDsol (5:4:1). The suspension was sonicated for 10 s and filtrated through 0.22 um Whatman filters.
